# Olfactory responses of the variegated fruit fly, *Phortica variegata*, an emerging vector of the zoonotic eyeworm *Thelazia callipaeda*, to ecologically relevant volatiles

**DOI:** 10.1186/s13071-025-06850-8

**Published:** 2025-06-02

**Authors:** Anna Laura Erdei, Magdolna Olívia Szelényi, Ferenc Deutsch, Balázs Kiss, Béla Péter Molnár

**Affiliations:** 1 Department of Plant Protection Biology, Chemical Ecology Unit, Swedish University of Agricultural Sciences, 23422 Lomma, Sweden; 2 Department of Chemical Ecology, Plant Protection Institute, HUN-REN Centre for Agricultural Research, Matronvásár, Hungary

**Keywords:** Dipteran vector, Olfactory responses, Chemical ecology, GC-EAD, Sexual olfactory dimorphism

## Abstract

**Background:**

The variegated fruit fly, *Phortica variegata* (Drosophilidae: Steganinae), is native to Europe and has emerged as a major vector of ocular nematosis caused by *Thelazia callipaeda* (Rhabditida: Thelaziidae), following the its introduction into Europe from Asia. Male *P. variegata* transmit these nematodes by feeding on tears of mammals, including wild and domestic carnivores (foxes, beech martens, wild cats, and dogs), lagomorphs, and humans. Understanding the olfactory responses of *P. variegata* to volatile cues is essential for developing attractant-based surveillance and control strategies, yet its olfactory ecology remains largely unexplored.

**Methods:**

We used gas chromatography coupled electroantennography to measure antennal responses to synthetic and natural volatile blends. A comparative analysis was performed on the antennal responses of both sexes of *P. variegata* and its well-studied relative, *Drosophila melanogaster*. Components of the synthetic blends were selected based on the odorant receptor repertoire of *D. melanogaster* and established mosquito attractants, with the rationale that conserved olfactory receptors among dipterans may allow *P. variegata* to detect similar compounds. Volatile extracts collected using active carbon adsorbent traps were also tested on the antennae and analyzed using gas chromatography coupled mass spectrometry.

**Results:**

Male *P. variegata* showed higher antennal responses to phenol, 3-octanone, and sulcatone than females, indicating olfactory sexual dimorphism. Compared to *D. melanogaster*, the antennae of *P. variegata* did not respond to several common plant alcohols and terpenoids. Instead, they showed stronger responses to compounds such as anisole, ethyl propanoate, butyl propanoate, propyl acetate, 3-octanone, nonanal, and decanal, suggesting that peripheral olfaction in *P. variegata* may be more tuned to microbial volatiles.

**Conclusions:**

*Phortica variegata* exhibits sexual dimorphism in olfactory responsivity, with males showing greater responsiveness to volatiles associated with host-seeking in other zoophilic dipterans, potentially guiding them to mammalian hosts for tear-feeding. Compared to *D. melanogaster*, *P. variegata* is more responsive to microbial and yeast-related volatiles and less responsive to plant-derived terpenoids, suggesting a foraging ecology linked to microbial substrates. The antennally detected volatiles identified in this study can be used as candidates for further behavioral studies to develop lures for vector management.

**Graphical Abstract:**

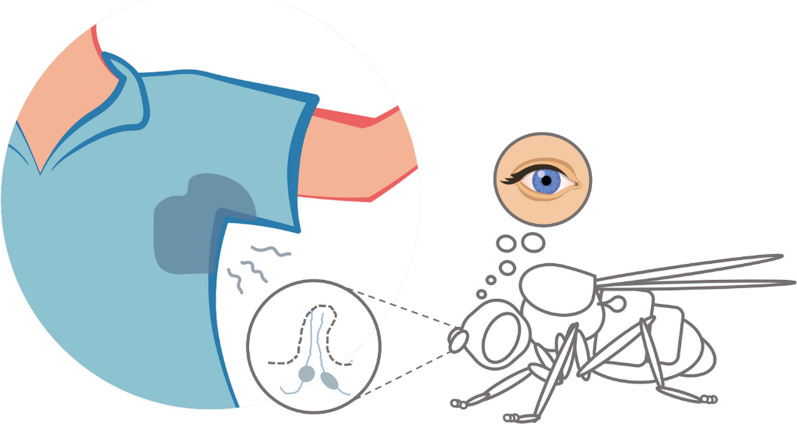

**Supplementary Information:**

The online version contains supplementary material available at 10.1186/s13071-025-06850-8.

## Background

Drosophilid fruit flies are undoubtedly among the most studied organisms in the world. However, due to their lack of agricultural and veterinary importance, the fungivorous and zoophilic flies of the closely related subfamily Steganinae (Drosophilidae) have received little interest so far. This perspective began to change when the role of *Phortica* species in the transmission of ocular nematosis was recognized.

*Phortica variegata* is native to Europe [1] and is known to feed on the tears of wild and domestic carnivores (foxes, beech martens, wild cats, and dogs), lagomorphs, and humans [2]. Over the past decade, ocular nematosis caused by *Thelazia callipaeda* (also called oriental eyeworm, Rhabditida, Thelaziidiiae), an ocular nematode native to Southeast Asia [3], has sharply increased and has become a growing public health concern in Europe.

*Phortica variegata* became an important vector of *T. callipaeda* when the nematode arrived in Europe. In its native habitat, *T. callipaeda* is mainly transmitted by other species of the genus *Phortica*, such as *P. magna* and *P. okadaii*. In Europe, however, *P. variegata* has emerged as the main vector [4]. Climate change and warmer winters have expanded the range and activity periods of this species by supporting overwintering and thereby facilitating the spread of *T. callipaeda* as well [5, 6]. Larvae of several *Phortica* species have been found to develop in fermenting tree sap [7, 8]. The distribution of *P. variegata* is associated with oak forests, and it has been shown that a chestnut-based rearing medium is suitable for larval development [9]. In his study of dipteran guilds utilizing small-scale forest food resources, Papp [10] found that adult *P. variegata* could be collected around fox feces and rotting fungi, suggesting that such microhabitats may serve as sites for mating, oviposition, or feeding. Additionally, larvae of other species in this subfamily were shown to develop on decaying plant matter and fungal substrates [8], indicating *P. variegata* may also use similar substrates as breeding sites. Little is known about the vectoring behavior, apart from the fact that *T. callipaeda* is only found in males and that females were not observed to feed on tears [11]. This behavioral dimorphism is in contrast with all other known vector insects, where zoophagy and vectoring are either entirely female-linked or exhibited by both sexes [11].

Insects rely primarily on the sense of smell to find food, mates, and oviposition sites in their complex environment. Their olfactory system is tuned to filter out “background noise” and detect volatile signals relevant for host recognition [12]. Chemical ecological research on vector insect olfaction supports control solutions by revealing the olfactory cues that modulate vector behavior. The identification of attractants and repellents can lead to new vector control solutions such as repellent formulations and baits for monitoring and mass trapping.

*Phortica variegata* belongs to the Drosophilidae family, where adaptation to new food sources was shown to be reflected in corresponding changes in the olfactory system; *Drosophila melanogaster*, a species feeding and ovipositing on overripe fruits, has high sensitivity for fermentation volatiles [7, 13]; herbivorous *Scaptomyza flava* has reduced sensitivity to those and increased sensitivity to leaf volatiles [14, 15]. *Drosophila sechellia* feeding on toxic Morinda fruits is attracted to short-chain fatty acids found in these fruits that repel other drosophilids [16].

Both sexes of *P. variegata* can be captured with fruit baits and vinegar and wine baits similar to *D. melanogaster* [17–19], although most flies caught with these baits are females, while those caught around the eyes are exclusively males [4, 17]. It was recently shown that supplementing vinegar wine baits with carvacrol abolishes this attraction [19]. Although currently there is limited information available on the olfactory repertoire of species belonging to the Steganinae subfamily, it can be hypothesized that the zoophilic behavior exhibited by *P. variegata* and the indicated association with microbial substrates can be accompanied by adaptations of the olfactory system.

Although no other known drosophilid species exhibit attraction to mammalian hosts, a useful parallel can be drawn to mosquitoes and tsetse flies, which are among the most extensively studied insect vectors. The evolution of zoophily in mosquitoes has been directly linked to olfactory adaptations. Host-seeking female mosquitoes rely on a heightened sensitivity to mammalian body odors [20–24]. l-lactic acid attracts *Aedes aegypti* females and has been shown to be involved in human discrimination, as it is more abundant in human sweat than in that of other mammals [20, 21]. It was also shown that an increased sensitivity to sulcatone is linked to human preference in *A. aegypti* where the increased expression of a sulcatone-sensitive odorant receptor, AaegOr4, supports host discrimination [24].

Additionally, short-chain saturated aldehydes in skin emissions [22, 23] and fluctuating CO_2_ levels, which enhance *A. aegypti*’s sensitivity to human odors [25, 26], have been shown to play key roles in identifying mammalian hosts. These findings in mosquitoes and the well-described olfactory adaptation in other drosophilid species suggest that similar mechanisms may underlie the zoophilic behavior observed in *P. variegata*, where olfactory adaptations could facilitate host recognition.

Currently, no efficient or species-specific attractants or repellents are available for *P. variegata*, posing a challenge for developing targeted vector control strategies. To our knowledge, the chemical ecology of *P. variegata*, or any species within the Steganinae subfamily, remains entirely unexplored. However, the recent annotation of odorant receptor genes in the *P. variegata* genome [27] opens the possibility to study olfactory mechanisms underlying tear feeding behavior. Given its attraction to mammals and fermentation-based baits, we hypothesized that *P. variegata* may rely on olfactory cues similar to those used by both mosquitoes and drosophilids to identify mammalian hosts and suitable microbial habitats. To investigate these hypotheses, we conducted olfactory recordings survey of *P. variegata* using gas chromatography coupled with electroantennographic detection (GC-EAD) comparing the response profile of both sexes to that of *D. melanogaster* using a panel of selected synthetic compounds and identified volatile components from host-related volatile sources and fermentation baits that are detected by the antennae of this species.

## Methods

### Collection of experimental animals and taxonomic identification

*Phortica variegata* males were collected by netting around the eyes of human collectors using hand-held aquarium nets in forest habitats around the outskirts of Budapest, Hungary, at Ördögárok (47.54254° N, 18.94572° E), Iluska spring (47.64546° N, 18.86779° E) and Piliscsaba (47.63866° N, 18.85353°E) during the early afternoon, at temperatures around 19 °C ± 8 °C. Females were captured using apple cider vinegar-baited live traps at the same locations. Animals were transferred into closed but not air-tight humidified vials using an aspirator. Species-level identification was based on the morphological taxonomic keys described by Bächli et al. [28] in The Drosophilidae (Diptera) of Fennoscandia and Denmark using methods identical to those of Kerezsi et al. [29]. Adults were sexed and placed individually in glass jars where access to apple fruit slices as food source was provided. The adults were kept on a 16/8 light cycle at room temperature (25 ± 2 °C) and at a relative humidity of 50 ± 5%. The wild-type *D. melanogaster* specimens used in the experiments were reared on a modified sugar-yeast-corn meal diet [30] under the same environmental conditions as described above.

### Preparation of synthetic mixtures

A preliminary comparison of antennal sensitivities of the two fruit fly species was done by testing synthetic mixtures using electroantennography coupled gas chromatography. The 47 components included in the mixtures (Table S1) were selected based on known ligands of *D*. *melanogaster* odorant receptors listed in DoOR, the online database of *D*. *melanogaster* odorant receptor responses [31], as well as on reported attractants involved in host-seeking behavior of blood-feeding vector insects such as mosquitoes and tsetse flies [32–40]. All compounds were diluted in HPLC grade hexane to a concentration of 100 ng/µl, and recordings with only solvent injected were also conducted as control. The purity and manufacturers of synthetic compounds are listed in Table S1.

### Volatile collection methods

Fresh fecal samples were collected from red fox (*Vulpes vulpes*), brown bear (*Ursus arctos*) and red deer (*Cervus elaphus*) in the Budakeszi Wildlife Park in Hungary. Human body odor was sampled from two female volunteers (volunteers did not use scented soap or deodorant 24 h prior to volatile sampling). The armpits and upper arms of the volunteers were rubbed with medical gauze for 5 min, which was later used to collect volatiles.

For wine-apple cider vinegar (W + ACV) headspace sampling, 2 dl of apple cider vinegar and 1 dl of red wine were mixed in a glass beaker. The samples were placed in oven bags (35 × 43 cm, Hewa). Volatile sampling was performed in the laboratory at room temperature. The incoming air was filtered by a carbon air inlet. The air stream was drawn from the oven bag through an activated carbon volatile trap (5 mg cartridge, Brechbühler AG, Switzerland) at a flow rate of 500 ml/min for 4 h.

The volatile traps were eluted with 200 μl  methylene chloride (Sigma Aldrich, HPLC grade). Prior to volatile collection, the active carbon airstream filters were cleaned at 200 °C, and the volatile traps were cleaned in a series of 2 ml methanol, 2 ml acetone, 2 ml hexane and 2 ml methylene chloride and heated up to 100 °C for 12 h. The volatile sampling was launched within 40 min of collecting the fecal and human body odor samples.

### Gas chromatography coupled mass spectrometry

The volatile extracts were analyzed by gas chromatography coupled with mass spectrometry (GC-MS, Agilent 6890 GC and 5975 MS, Agilent Technologies) equipped with a HP-5 UI capillary column (30 m × 0.25 mm × 0.25 µm, J&W Scientific, Folsom, CA, USA); 2 µl of volatile extracts was auto-injected into the split/splitless injection port operated in splitless mode heated to 270 °C using a 1-min splitless time. Helium was used as a carrier gas with a flow rate of 1 ml/min. The initial oven temperature was held at 50 °C for 1 min and then increased by 10 °C/min to 270 °C. The final temperature was held for 10 min. The mass spectrometer source was operating at 250 °C in electron ionization mode at 70 eV and the detector scanned in the 29–300 m/z range.

The GC-MS results were analyzed using Agilent Mass Hunter B.08.00, and the peaks were manually integrated. Compounds were tentatively identified by matching their mass spectra with those found in MS Libraries (NIST21 and Wiley12). Identifications were also verified by comparing calculated Kováts indices (KI) using C8-C20 alkane calibration standard to those found in NIST WebBook database, where only references using authentic standards were considered, and key compounds were also identified using authentic synthetic standards (Table S2).

### Gas chromatography coupled electroantennography

The biologically active components of volatile extracts were identified using gas chromatography coupled electroantennographic detection (GC-EAD). The Agilent 6890 N gas chromatograph (GC) was equipped with an HP-5 UI capillary column (30 m × 0.32 mm × 0.25 µm, J&W Scientific, Folsom, CA, USA); 2 µl of volatile extracts was manually injected into the injection port of the gas chromatograph, operated in splitless mode and heated to 230 °C using a 1-min splitless time. The carrier gas was helium, and the column flow was 4 ml/min. The initial oven temperature was held at 50 °C for 1 min and then in the first ramp increased by 10 °C/min to 270 °C and in the second ramp by 30 °C/min to 230 °C. The final temperature was held for 5 min.

The GC effluent was split in a low dead volume Graphpack 3D/2 four-way splitter. Two non-coated deactivated fused silica capillary columns (100 cm × 0.32 mm) were connected to the four-way splitter. One led to the flame ionization detector (FID) heated to 300 °C, and the other line was fitted into a transfer line heated to 235 °C (Syntech, Kirchzarten, Germany). The capillary column protruded from the heated transfer line into an inert glass tube (10 mm I.D.) that had a charcoal-filtered and humidified airflow of 1 l/min transferring the effluent over the antennal preparation.

Female and male adults of *P. variegata* and *D. melanogaster* were immobilized in 200-µl pipette tips. The tip of the pipette was cut, and the animal was pushed forward until half of the eye was uncovered in the pipette tip, but the proboscis was still covered. The silver/silver chloride electrodes were immersed in Ringer solution in two finely pulled glass capillaries. The glass capillary of the reference electrode was inserted into one of the eyes of the restrained fly, and the glass capillary covering the recording electrode was pushed to hold firm contact with the dorsomedial region of the funiculus. The antennal signal was amplified 10 times, and the analog–digital conversion was done by IDAC-2 (Syntech). The recording was done simultaneously with the FID signal using GC-EAD software (GC-EAD 2014, vers. 1.2.5, Syntech). The eight synthetic mixtures were tested sequentially on the same individuals in randomized order. Before each GC-EAD run, a glass Pasteur pipette loaded with 100 ng 1-hexanol in 10 µl mineral oil was used as a test stimulus to assess the quality of preparation and electric contact. The synthetic mixes were tested on at least three specimens of both species and both sexes. The volatile extracts were tested on male *P. variegata* specimens, with the exception of fox feces, which was tested on both females and males.

### Data processing and statistical analysis

Statistical analysis and data visualization were performed using R (v. 4.2.0) in RStudio (RStudio Team (2023 v. 6.0.421). Similarities and dissimilarities between the antennal response profile of *D*. *melanogaster* and *P*. *variegata* individuals to synthetic compounds were investigated by principal coordinates analysis (PCoA) using the *capscale* function and by non-metric multidimensional scaling (NMDS) of the *vegan* package (v. 2.6–4) [41] using Jaccard dissimilarity as a distance measure on non-binary data. The responses were standardized across individuals by dividing the responses by the average of responses for the individual. The responses were also standardized across each compound by Z-scoring: the average response to the compound is subtracted from the response of each individual and divided by the standard deviation of responses to the compound. Permutational multivariate analysis of variance (PERMANOVA) was performed on the Jaccard dissimilarity as a distance measure to compare groups using *adonis2* function of the *vegan* package (v. 2.6–4) [41], and the significance values for multiple comparisons were adjusted by Benjamini-Hochberg correction (*P*-value adj). Prior to PERMANOVA, permutational multivariate analysis of dispersion (PERMDISP) was performed using the *betadisper* function to verify dispersion homogeneity between groups.

**Fig. 1:**
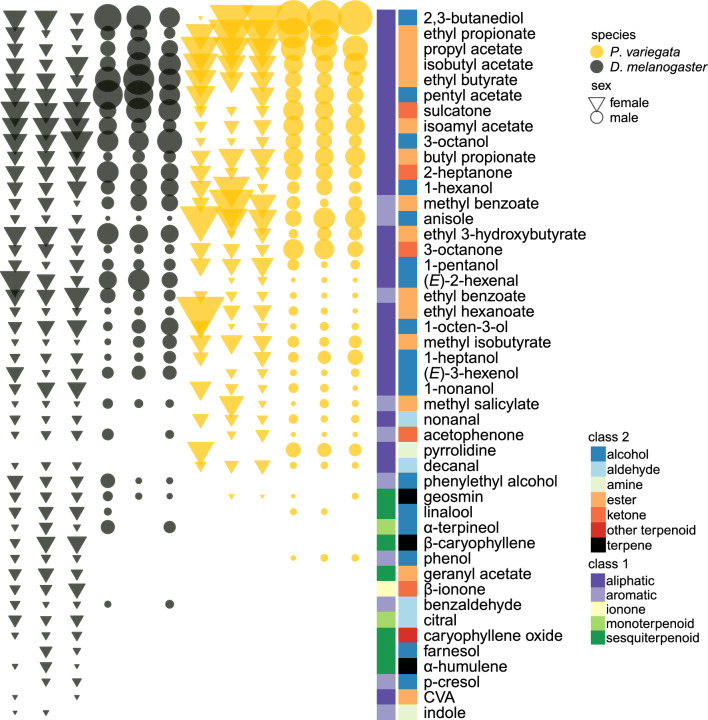
The antennal sensitivity of *Drosophila melanogaster* and *Phortica variegata* to a panel of synthetic compounds was measured using GC-EAD. The responses were normalized by individuals by dividing each antennal response with the average of responses for the individual. The radius of symbols represents the relative response size. Color codes indicate the chemical classes of the selected synthetic compounds

**Fig. 2:**
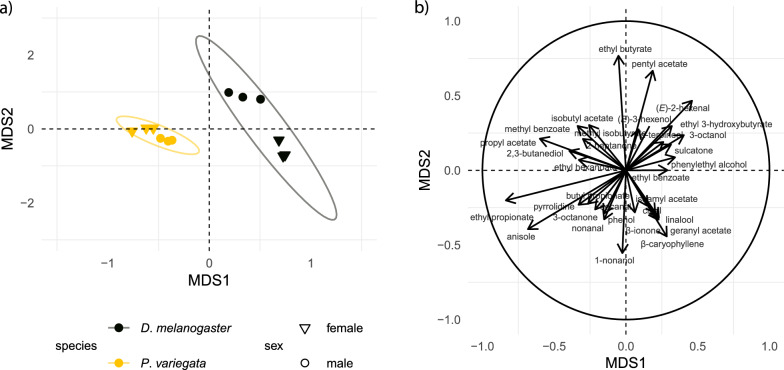
Principal coordinate analysis (PCoA) based on the relative amplitude of antennal responses of *Drosophila melanogaster* and *Phortica variegata* individuals to synthetic volatiles. **a** Clustering of individuals along MDS1 and MDS2. The relative corrected eigenvalues denoting the percentage contribution of each axis to the total variation is 0.58 for MDS1 and 0.25 for MDS2. **b** The length and direction of vectors on the biplot show the contribution of individual responses to the separation along MDS1 and MDS 2. Only compounds with eigenvalues < −0.3 or > 0.3 are plotted

**Fig. 3:**
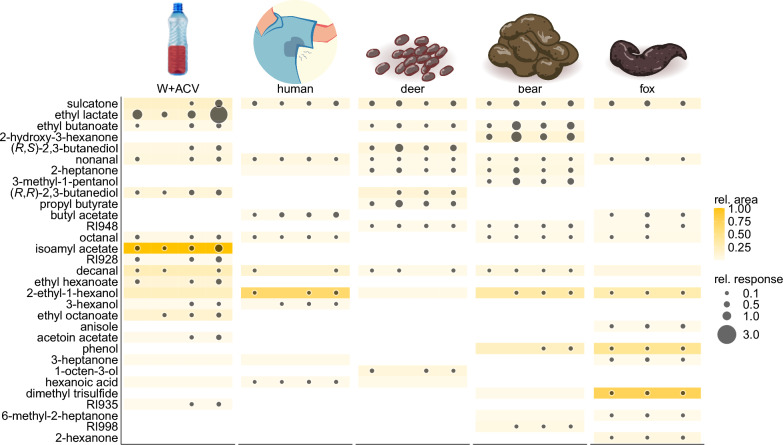
The antennal responses of male *Phortica variegata* to components of a volatile sample of red wine-apple cider vinegar bottle traps (W + ACV) and ecologically relevant volatile blends (human body odor, deer, bear, and fox feces). The relative peak areas of compounds are shown using a color scale, whereas response sizes are shown as the radii of circles

**Fig. 4:**
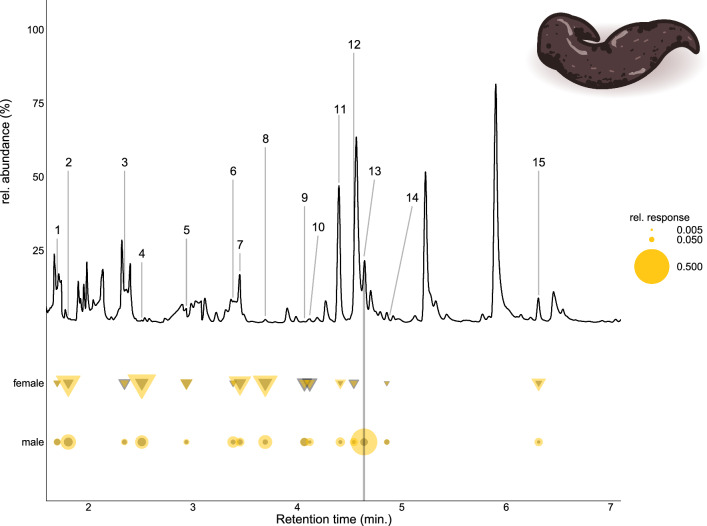
Comparison of female and male antennal responses of *Phortica variegata* to fox feces volatiles. Section of the FID chromatogram of the fox feces volatile sample is plotted against the average responses of *P. variegata* individuals. The numbers represent the following active components: (1) RI738, (2) RI748, (3) 2-hexanone, (4) butyl acetate, (5) RI853, (6) 3-heptanone, (7) RI900, (8) anisole, (9) RI948, (10) 6-methyl-2-heptanone, (11) dimethyl trisulfide, (12) phenol, (13) sulcatone, (14) octanal (15) nonanal. The standardized amplitude of average antennal response is visualized by the radius of yellow dots; the gray dots show the standard deviation of standardized antennal responses. Vertical grey line shows the missing response in females to (13) sulcatone

To identify gender- and species-specific responses to individual compounds, we used multi-level pattern analysis with the *multipatt* function of *indicspecies* package [42] and adjusted *P*-values using Benjamini-Hochberg correction using the *p.adjust* function from the *stats* package. All figures were visualized using geom_point (Figs. 1, 2, 3, 4), geom_segment (Fig. 2), geom_tile (Fig. 3), and geom_line (Fig. 4) functions of the *ggplot2* v.3.5.1 [43] package.

## Results

We first assessed differences in antennal responses between *P. variegata* and *D. melanogaster* to synthetic compounds. These were selected based on their detection by olfactory receptors of *D. melanogaster* (as listed in the DoOR database [31]) as well as on their known roles in modulating host-seeking behavior in blood-feeding dipterans, such as mosquitoes and tsetse flies [32–40]. Both species were sensitive to aliphatic esters. However, *P. variegata* showed no antennal responses to the monoterpenoids and sesquiterpenoids tested, with the exception of linalool (Fig. 1, Fig. S1).

PCoA (Fig. 2) and NMDS (Fig. S2) were applied using non-binary Jaccard dissimilarity to assess divergence in antennal response profiles. Individuals from the same species clustered closely together, whereas *P. variegata* and *D. melanogaster* separated along MDS1. The eigenvalues for MDS1 (0.58) and MDS2 (0.25) indicate that MDS1 explained most of the variation. After verifying the assumption of dispersion homogeneity (*P*-value_PERMDISP_ = 0.27), PERMANOVA confirmed significant divergence in response profiles between species (F₁ = 7.386, *P*-value_PERMANOVA_ = 0.002). Pairwise comparisons with Benjamini-Hochberg correction also supported this difference (*P*-value adj = 0.004).

The multi-level pattern comparison (Table S3) showed that *P. variegata* had higher relative responses to propyl acetate, ethyl propanoate, butyl propanoate, anisole, 3-octanone, nonanal, and decanal than *D. melanogaster*. *Phortica variegata* males had higher responses to phenol, 3-octanone, and sulcatone (6-methyl-5-hepten-2-one) than females. Although males exhibited numerically higher responses to nonanal and decanal, this difference was not statistically significant (*P*-value adj > 0.05).

Given that adult male *P. variegata* are captured in fermentation baits, associated with mammalian feces, and attracted to mammals, further GC-EAD recordings were performed using ecologically relevant volatile samples, including W + ACV; feces from bear, deer, and fox; and human body odor. Electrophysiological recordings revealed antennal responses to 31 volatile components (Fig. 3).

The detected components of mammalian samples closely resembled those of W + ACV (Table S4), as evidenced by similar antennal response profiles (Fig. 3). Ethyl lactate and isoamyl acetate were detected exclusively in W + ACV volatiles and elicited antennal responses. Phenol and dimethyl trisulfide were only present and were sensed in the bear and fox feces samples. Nonanal, sulcatone, and decanal were components of all samples (Table S4). Nonanal and sulcatone elicited antennal response in all volatile samples, while decanal did not elicit antennal responses in fox feces volatiles, likely because of its low abundance (Fig. 3). Anisole and hexanoic acid elicited antennal responses but the former was only present in the volatile sample of fox feces and the latter in human body odor samples.

Since *P. variegata* has been found in fox faeces [10], we tested whether antennal responses of sexes differ from volatiles emitted from fox feces (Fig. 4). Sulcatone failed to elicit antennal responses in females, but relative response amplitudes to other volatiles did not differ significantly between sexes (Fig. 4).

## Discussion

We compared antennal responses of *P. variegata* and *D. melanogaster* to a panel of synthetic odorants known to activate olfactory receptors in *D. melanogaster* and influence host-seeking in other mammal-attracted dipterans. While both species responded to aliphatic esters, *P. variegata* showed limited responses to monoterpenoids and sesquiterpenoids, and multivariate analyses revealed clear species- and sex-related divergence in response profiles.

The behavioral dimorphism, in which only male flies feed on tears, may also be reflected in sex-specific olfactory sensitivity, similar to *A. aegypti* where females express a higher number of ORs compared to males, likely linked to their need to recognize hosts for blood feeding [44]. To test this hypothesis, we compared the antennal response of female and male *P. variegata* using a panel of synthetic compounds (Figs. 1, 2). Multi-level pattern analysis revealed significant differences in response amplitudes between the sexes (Table S3).

The antenna of males exhibited significantly higher responses to synthetic phenol, 3-octanone, and sulcatone. Sulcatone was found in all tested relevant volatile samples (Fig. 3). Moreover, while sulcatone elicited responses in synthetic blends, it showed a diminished antennal response in females when derived from fox feces (Fig. 4). These results could be explained by the higher antennal sensitivity of male *Phortica* flies, as the amount of sulcatone in fox feces was possibly below the detection threshold for females. As sulcatone is abundant in human skin and animal emissions [45, 46], a heightened sensitivity might allow males to detect and locate mammalian hosts more effectively than females. Sexual dimorphism in peripheral olfaction is a well-documented phenomenon in insects, often reflected in differences in antennal structure, the number of odorant receptors, and sensitivity to specific compounds [47–49]. For instance, sexual dimorphism in olfaction of vector insects has been observed in *A. aegypti* [50], *Culex pipiens quinquefasciatus* [51], and *Anopheles gambiae* [47]. Notably, *A. aegypti* strains that prefer human hosts over cattle have been shown to overexpress the odorant receptor AaegOr4, which has a high affinity for sulcatone [24]. However, no significant sexual dimorphism was observed in the expression levels of this receptor [44], and the antennal sensitivity to sulcatone has not been compared yet. To determine whether male *P. variegata* files are more sensitive to sulcatone and whether this compound is important in host-seeking, further studies involving dose-dependency measurements and behavioral experiments are needed.

Differences observed for phenol in synthetic blends were not significant in fox feces samples, likely because of dose dependency, as the fox feces samples were more concentrated than the synthetic blends tested based on the comparison of FID traces. This observation again highlights the importance of considering dose dependency when evaluating olfactory responses and underscores the need for carefully controlled experiments to accurately assess sex-specific differences in chemosensation and behavior, as demonstrated for both sulcatone and phenol with female *Culicoides nubeculosus* where attraction and repellence are strictly dose-dependent [46]. The observed sensory differences between sexes of *P. variegata* could explain the behavioral dimorphism, but further experiments are needed to establish a causal link between the detection of these compounds and their effect on behavior.

Species-specific differences were observed between the response profiles of *D*. *melanogaster* and *P. variegata* to synthetic compounds as they separated along the first axis of PCoA (Fig. 1). For several other compounds, a dose-dependent response was observed in the ecologically relevant volatiles samples (Fig. 3). Compared to *D. melanogaster*, both sexes of *P. variegata* showed an increased sensitivity to anisole (methoxybenzene), which is the main constituent of anise seed essential oil [52], and were described to be present in essential oils prepared from other plants [53]. Intriguingly, anisole is also emitted from decomposing leaf litter, such as that of poplar [54], and can be emitted by microbes such as *Penicillium expansum* during the degradation of lignin [55]. It was reported that *P. variegata* adults feed on fermenting tree sap [8, 56], which might be a rich source of anisole and related methoxybenzenes. Surprisingly, anisole was also a minor component of fox feces headspace, and both sexes of *P. variegata* responded to this component of the volatile extracts.

The antennae of *P. variegata* were more responsive than those of *D. melanogaster* to several common volatile compounds emitted from fermented substrates, including ethyl and butyl propanoate, propyl acetate, 3-octanone, nonanal, and decanal. These compounds are found in a wide range of natural sources. Nonanal and decanal are also major components of human body odor, and they were shown to be attractive to *Culex* mosquitoes [57], while the high ratio of these compounds was shown to decrease the attraction of *A. aegypti* to human body emissions [23]. Several aliphatic esters from the synthetic blend are often associated with fermenting plant materials, yeasts, and ripening fruits [58–60], and similar to *D. melanogaster* antennae, those of *P. variegata* responded to isoamyl and isobutyl acetate and had a significantly higher responsivity to ethyl- and butyl propanoate and propyl acetate.

Interestingly, *P. variegata* exhibited a weaker response to (*E*)−2-hexenal and (*E*)−3-hexenol, which are characteristic green leaf volatiles emitted upon mechanical damage of plant tissues [61]. These compounds are repellent for *D. melanogaster* and were hypothesized to be related to the discrimination of ripe fruits from ripening ones that are unsuitable for oviposition [62]. Furthermore, *P. variegata* antennae did not respond to terpenoids selected from the DOOR, except males showing a weak response to linalool. β-caryophyllene, farnesol, and α-humulene are common sesquiterpenoid compounds in plant volatile emissions [63]. β-caryophyllene, α-terpineol, and α-humulene are major ligands of OR19a expressed in trichoid sensilla [64], and farnesol is a major ligand of OR83c expressed in intermediate sensilla [65] on the antennae of *D. melanogaster*. Several other ORs such as OR69a are also involved in the detection of terpenoids. Bastide et al. [27] identified two orthologs of OR19a and one of OR83c in the genome of *P. variegata*. However, the functionality of these genes, their expression pattern, and their main ligands are currently unknown.

Since terpenoids are detected by multiple odorant receptors in *D. melanogaster*, the lack of response in *P. variegata* to several terpenoids may reflect ecological differences between the species, suggesting that *P. variegata* relies less on the identification of plant-derived resources than *D. melanogaster*.

The lower sensitivity of *P. variegata* to several ubiquitous plant volatile compounds compared to *D. melanogaster* and increased sensitivity to several volatile compounds common in microbial volatile emissions indicate that fungal and microbial substrates might be more important in the ecology of this species compared to *D. melanogaster*. According to our current knowledge, many species in this group are associated with fungi or feeding on decaying plant material. Similarly to the attraction of mosquitoes to their hosts [66, 67], the attraction of *P. variegata* to mammalian hosts can be based on otherwise common microbial volatiles combined with carbon dioxide or visual cues.

Based on this first report on the olfactory responses of *P. variegata*, the behavioral significance of ethyl and butyl propanoate, propyl acetate, 3-octanone, nonanal, decanal, and sulcatone for females and males should be further evaluated in laboratory and field behavioral bioassays. The identification of new attractants can provide a basis for developing both monitoring and mass trapping solutions for the future management of this vector species.

## Conclusions

Our study demonstrates that *P*. *variegata* exhibits sexual dimorphism in olfactory responsivity, with males showing increased responsivity to specific volatiles such as sulcatone, phenol, and 3-octanone, which may help them locate mammalian hosts, aligning with their behavioral dimorphism in feeding on tears. Additionally, the comparative olfactory analysis with *D. melanogaster* revealed that *P. variegata* shows a stronger responsivity to several microbial and yeast-related volatiles and a strongly reduced responsivity to common plant volatile terpenoids, reinforcing the idea that its foraging ecology differs from that of *D. melanogaster* and fungal and microbial substrates might be ecologically even more relevant for this species. The study highlights several antennally active volatiles that could be assessed in field and laboratory behavioral experiments to investigate their ecological roles and to potentially use them to develop monitoring and control strategies against this dipteran vector species.

## Supplementary Information

Additional file 1: Fig. S1. Heatmap of raw responses comparing  *D. melanogaster* and *P. variegata* individuals to the panel of tested synthetic volatiles. 

Additional file 2: Fig. S2. Non-metric multidimensional scaling based on the antennal responses of *D. melanogaster* and *P. variegata* individuals to the panel of tested synthetic volatiles. 

Additional file 3: Table S1. The producer and purity of synthetic compounds selected for GC-EAD testing on *D. melanogaster* and* P. variegata*. 

Additional file 4: Table S2. Calculated linear retention indices (RI calc.) and reference retention indices (RI lib.)  from literature data on non-polar HP-5 capillary column.

Additional file 5: Table S3. Results of multilevel pattern analysis for comparing antennal responses to synthetic compounds between *D. melanogaster* and *P. variegata* females and males.  The *P*-value was adjusted (*P*-value adj.) using Benjamini-Hochberg adjustment. The column pattern shows which species and sex combinations showed significantly higher relative responses.

Additional file 6: Table S4. The composition of volatile samples tested using gas chromatography with electroantennographic recording. The relative abundance of each component was calculated as a percentage compared to the component with the highest peak area.

## Data Availability

Data utilized in this manuscript, along with the scripts to generate the figures, are available at 10.6084/m9.figshare.28920173.

## Abbreviations

*A. aegypti*: *Aedes aegypti*
*D. melanogaster*: *Drosophila melanogaster*
GC-MS: Gas chromatography-mass spectrometry
GC-EAD: Gas chromatography-electroantennography
OR: Odorant receptor
NMDS: Non-metric multidimensional scaling
PcoA: Principal coordinate analysis
*P. variegata*: *Phortica variegata*
