# Supplementary material for: Olfactory responses of the variegated fruit fly, Phortica variegata, an emerging vector of the zoonotic eyeworm Thelazia callipaeda, to ecologically relevant volatiles
Source: Parasit Vectors. 2025 Jun 2;18:204. doi: 10.1186/s13071-025-06850-8 (PMC12131565; doi:10.1186/s13071-025-06850-8)
Supplement: Supplementary file 2 — Additional file 2: Fig. S2. Non-metric multidimensional scaling based on the antennal responses of D. melanogaster and P. variegata individuals to the panel of tested synthetic volatiles. [file 13071_2025_6850_MOESM2_ESM.docx]

**
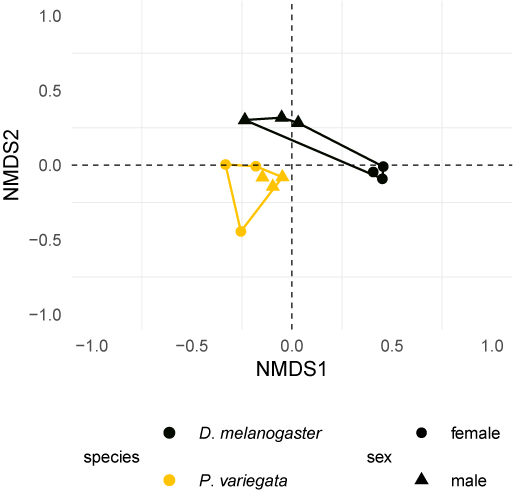
**

**Fig S2.** Non-metric multidimensional scaling based on the antennal responses of *D. melanogaster* and *P. variegata* individuals to the panel of tested synthetic volatiles.
