# Supplementary material for: Olfactory responses of the variegated fruit fly, Phortica variegata, an emerging vector of the zoonotic eyeworm Thelazia callipaeda, to ecologically relevant volatiles
Source: Parasit Vectors. 2025 Jun 2;18:204. doi: 10.1186/s13071-025-06850-8 (PMC12131565; doi:10.1186/s13071-025-06850-8)
Supplement: Supplementary file 3 — Additional file 3: Table S1. The producer and purity of synthetic compounds selected for GC-EAD testing on D. melanogaster and P. variegata. [file 13071_2025_6850_MOESM3_ESM.docx]

**Table S1.** Synthetic compounds selected for GC-EAD testing on *D. melanogaster* and *P. variegata*. The purity and producers as well as the reference literature that were used in the selection procedure and the most sensitive *D. melanogaster* odorant receptors are listed.

| **compound** | **CAS** | **purity** | **producer** | **odorant receptor** | **reference** |
| --- | --- | --- | --- | --- | --- |
| methyl-isobutyrate | 547-63-7 | 99% | Sigma Aldrich | 22a, 42b, 59b | 1. |
| propyl acetate | 109-64-4 | 98% | Sigma Aldrich | 42a, 43b,47a, 42b | 1. |
| geosmin | 16423-19-1 | 99% | Sigma Aldrich | 56a | 1., 2. |
| ethyl butyrate | 105-54-4 | 98% | Sigma Aldrich | 42a, 43b, 42b, 85a | 1. |
| ethyl benzoate | 93-89-0 | 99% | Sigma Aldrich | 98a, 10a, 69a, 67a | 1. |
| 4-methylphenol | 106-44-5 | 99% | Acros Organics | 71a, 30a, 69a | 1., 3. |
| 2-heptanone | 110-43-0 | 99% | Sigma Aldrich | 85c, 85b, 98a, 47a | 1. |
| 1-octen-3-ol | 3391-86-4 | 98% | Sigma Aldrich | 13a, 85c, 98a, 85b | 1., 4-7. |
| methyl salicylate | 119-36-8 | 99% | Sigma Aldrich | 10a, 69a, 71a, 1a | 1. |
| farnesol | 4602-84-0 | 95% | Sigma Aldrich | 83c | 1. |
| ethyl 3-hydroxybutyrate | 5405-41-4 | 98% | Sigma Aldrich | 13a , 69a, 22a, 92a | 1. |
| (E)-3-hexenol | 928-96-1 | 98% | Sigma Aldrich | 35a, 85b, 85a, 9a | 1. |
| acetophenone | 98-86-2 | 98% | Sigma Aldrich | 67b, 45b, 10a, 59a | 1., 8. |
| 2,3-butanediol | 513-85-9 | 99% | Fluka Chemicals | 9a, 92a, 43b, 69a | 1. |
| pyrrolidine | 123-75-1 | 99% | Sigma Aldrich | 85b, 65a, 88a | 1. |
| (E)-2-hexenal | 6728-26-3 | 98% | Sigma Aldrich | 7a , 35a, 42a, 67b | 1. |
| benzaldehyde | 100-52-7 | 99.50% | Sigma Aldrich | 45b , 67b, 30a, 7a | 1. |
| α-terpineol | 10482-56-1 | 90% | Sigma Aldrich | 69a , 19a | 1. |
| 3-octanol | 589-98-0 | 99% | Sigma Aldrich | 85c , 69a, 85b, 98a | 1., 5. |
| pentyl acetate | 628-63-7 | 99% | Sigma Aldrich | 47a , 85c, 98a, 35a | 1. |
| methyl benzoate | 93-58-3 | 99% | Sigma Aldrich | 98a , 10a, 67a, 71a | 1. |
| bornyl acetate | 5655-61-8 | 95% | Sigma Aldrich | 19a | 1. |
| 1-pentanol | 71-41-0 | 99% | Sigma Aldrich | 35a , 85b, 43b, 67c | 1. |
| 1-heptanol | 111-70-6 | 98% | Sigma Aldrich | 35a , 85c, 74a, 13a | 1., 6. |
| indole | 120-72-9 | 99% | Sigma Aldrich | 85b | 1., 9. |
| geranyl acetate | 105-87-3 | 97% | Sigma Aldrich | 82a , 69a, 83c, 98a | 1. |
| ethyl propionate | 105-37-3 | 99% | Sigma Aldrich | 42b , 43b, 22a, 59b | 1. |
| ethyl hexanoate | 123-66-0 | 99% | Sigma Aldrich | 22a, 47a, 67a, 85b | 1. |
| caryophyllene oxide | 1139-30-6 | 95% | Sigma Aldrich | 19a | 1. |
| butyl propionate | 0590-01-02 | 99% | Acros Organics | 67a , 19a, 47a, 42b | 1. |
| α-humulene | 6753-98-6 | 96% | Sigma Aldrich | 19a, 7a | 1. |
| 3-octanone | 106-68-3 | 99% | Sigma Aldrich | 83a, 19a | 1. |
| phenol | 108-95-2 | 99% | Sigma Aldrich | 7a | 1., 10. |
| linalool | 78-70-6 | 97% | Sigma Aldrich | 69a , 98a, 19a, 13a | 1. |
| isoamyl acetate | 123-92-2 | 99.50% | Sigma Aldrich | 43b, 98a, 47a, 22a | 1. |
| citral | 5392-40-5 | 95% | Sigma Aldrich | 83c | 1. |
| β-ionone | 79-77-6 | 96% | Sigma Aldrich | 69a | 1. |
| β-caryophyllene | 87-44-5 | 80% | Sigma Aldrich | 69, 13a | 1. |
| 1-nonanol | 0143-08-08 | 98% | Fluka Chemicals | 74a, 45a | 1., 6. |
| sulcatone | 110-93-0 | 99% | Sigma Aldrich | 85b , 98a, 67a, 13a | 1., 7., 11. |
| phenylethyl alcohol | 60-12-8 | 99% | Sigma Aldrich | 67b , 67a, 69b, 35a | 1. |
| nonanal | 124-19-6 | 95% | Sigma Aldrich | 69a, 7a | 1., 7. |
| isobutyl acetate | 110-19-0 | 98% | Sigma Aldrich | 43b, 98a, 47a, 22a | 1. |
| decanal | 112-31-2 | 98% | Sigma Aldrich | 69a , 67b, 10a | 1., 7. |
| (Z)-vaccenyl acetate | 6186-98-7 | 99% | Pherobank | 42a | 1. |
| anisole | 100-66-3 | 99.70% | Sigma Aldrich | 94a , 30a, 45b, 59a | 1. |
| 1-hexanol | 111-27-3 | 99% | Sigma Aldrich | 67b, 35a, 85c, 13a | 1. |

1. Münch, D., & Galizia, C. G. (2016). DoOR 2.0—Comprehensive mapping of Drosophila melanogaster odorant responses. Scientific Reports, 6, 21841.

2. Melo, N., Wolff, G. H., Costa-da-Silva, A. L., Arribas, R., Triana, M. F., Gugger, M et al. (2020). Geosmin attracts Aedes aegypti mosquitoes to oviposition sites. Current Biology, 30(1), 127-134.

3. Owaga, M. L., Hassanali, A., & McDowell, P. G. (1988). The role of 4-cresol and 3-n-propylphenol in the attraction of tsetse flies to buffalo urine. International Journal of Tropical Insect Science, 9(1), 95-100.

4. Takken, W., & Kline, D. L. (1989). Carbon dioxide and 1-octen-3-ol as mosquito attractants. Journal of the American Mosquito Control Association, 5(3), 311-316.

5. Vale, G. A., & Hall, D. R. (1985). The role of 1-octen-3-ol, acetone and carbon dioxide in the attraction of tsetse flies, Glossina spp.(Diptera: Glossinidae), to ox odour. Bulletin of Entomological Research, 75(2), 209-218.

6. Magalhães-Junior, J. T., Barrouin-Melo, S. M., Corrêa, A. G., da Rocha Silva, F. B., Machado, V. E., Govone, J. S., & Pinto, M. C. (2014). A laboratory evaluation of alcohols as attractants for the sandfly Lutzomyia longipalpis (Diptera: Psychodidae). Parasites & vectors 7, 1-5.

7. Dormont, L., Mulatier, M., Carrasco, D., & Cohuet, A. (2021). Mosquito attractants. Journal of chemical ecology, 47, 351-393.

8.Zhang, H., Zhu, Y., Liu, Z., Peng, Y., Peng, W., Tong, L. et. al (2022). A volatile from the skin microbiota of flavivirus-infected hosts promotes mosquito attractiveness. Cell, 185(14), 2510-2522.

9. Harraca, V., Syed, Z., & Guerin, P. M. (2009). Olfactory and behavioural responses of tsetse flies, Glossina spp., to rumen metabolites. Journal of Comparative Physiology A, 195, 815-824.

10. Vale, G. A., Hall, D. R., & Gough, A. J. E. (1988). The olfactory responses of tsetse flies, Glossina spp.(Diptera: Glossinidae), to phenols and urine in the field. Bulletin of Entomological Research, 78(2), 293-300.
