# Supplementary material for: Olfactory responses of the variegated fruit fly, Phortica variegata, an emerging vector of the zoonotic eyeworm Thelazia callipaeda, to ecologically relevant volatiles
Source: Parasit Vectors. 2025 Jun 2;18:204. doi: 10.1186/s13071-025-06850-8 (PMC12131565; doi:10.1186/s13071-025-06850-8)
Supplement: Supplementary file 4 — Additional file 4: Table S2. Calculated linear retention indices (RI calc.) and reference retention indices (RI lib.) from literature data on non-polar HP-5 capillary column. [file 13071_2025_6850_MOESM4_ESM.docx]

**Table S2.** Calculated linear retention indices (RI calc.) and reference retention indices (RI lib.) from literature data on non-polar HP-5 capillary column.

| compound | CAS | RI (mean) | RI (lib) | references |
| --- | --- | --- | --- | --- |
| dimethyl disulfide | 624-92-0 | 763 | 756 | Solina, M.; Baumgartner, P.; Johnson, R.L.; Whitfield, F.B., Volatile aroma components of soy protein isolate and acid-hydrolysed vegetable protein, Food Chem., 2005, 90, 4, 861-873, https://doi.org/10.1016/j.foodchem.2004.06.005 . |
| 3-methyl-2-pentanone | 565-61-7 | 768 | 759 | García, C.; Martín, A.; Timón, M.L.; Córdoba, J.J., Microbial populations and volatile compounds in the 'bone taint' spoilage of dry cured ham, Lett. Appl. Microbiol., 2000, 30, 1, 61-66, https://doi.org/10.1046/j.1472-765x.2000.00663.x . |
| 2-methyl-propanoic acid | 79-31-2 | 774 | 775 | Kotseridis, Y.; Baumes, R., Identification of impact odorants in Bordeaux red grape juice, in the commercial yeast used for its fermentation, and in the produced wine, J. Agric. Food Chem., 2000, 48, 2, 400-406, https://doi.org/10.1021/jf990565i . |
| 1-pentanol* | 71-41-0 | 774 | 776 | Mildner-Szkudlarz, S.; Jelen, H.H., The potential of different techniques for volatile compounds analysis coupled with PCA for the detection of the adulteration of olive oil with hazelnut oil, Food Chem., 2008, 110, 3, 751-761, https://doi.org/10.1016/j.foodchem.2008.02.053 . |
| toluene | 108-88-3 | 774 | 773 | Pino, J.A.; Mesa, J.; Muñoz, Y.; Martí, M.P.; Marbot, R., Volatile components from mango (Mangifera indica L.) cultivars, J. Agric. Food Chem., 2005, 53, 6, 2213-2223, https://doi.org/10.1021/jf0402633 . |
| ethyl 2-methylpropanoate | 97-62-1 | 771 | 770 | Lopez, R.; Ferreira, V.; Hernandez, P.; Cacho, J.F., Identification of impact odorants of young red wines made with Merlot, Cabernet Sauvignon and Grenache grape varieties: a comparative study, J. Sci. Food Agric., 1999, 79, 11, 1461-1467, https://doi.org/10.1002/(SICI)1097-0010(199908)79:11<1461::AID-JSFA388>3.0.CO;2-K . |
| 2-methyl-1-propanol | 110-19-0 | 783 | 781 | Shalit, M.; Katzir, N.; Tadmor, Y.; Larkov, O.; Burger, Y.; Shalekhet, F.; Lastochkin, E.; Ravid, U.; Amar, O.; Edelstein, M.; Karchi, Z.; Lewinsohn, E., Acetyl-CoA: alcohol acetyltransferase activity and aroma formation in ripening melon fruits, J. Agric. Food Chem., 2001, 49, 2, 794-799, https://doi.org/10.1021/jf001075p . |
| methyl 2-methylbutanoate | 868-57-5 | 786 | 780 | Pino, J.A.; Mesa, J.; Muñoz, Y.; Martí, M.P.; Marbot, R., Volatile components from mango (Mangifera indica L.) cultivars, J. Agric. Food Chem., 2005, 53, 6, 2213-2223, https://doi.org/10.1021/jf0402633 . |
| (*R,R*)-2,3-butanediol* | 513-85-9 | 787 | 784 | Xu, L.-L.; Han, T.; Wu, J.-Z.; Zhang, Q.-Y.; Zhang, H.; Huang, B.-K.; Rahman, K., Comparative research of chemical constituents, antifungal and antitumor properties of ether extracts of Panax ginseng and its endophytic fungus, 2012, retrieved from http://www.thefreelibrary.com/Comparative .... |
| 2,3-hexanedione | 3848-24-6 | 791 | 786 | Forero, M.D.; Quijano, C.E.; Pino, J.A., Volatile compounds of Chile pepper (Capsicum annuum L. var. glabriusculum) at two ripening stages, Flavour Fragr. J., 2008, 24, 1, 25-30, https://doi.org/10.1002/ffj.1913 . |
| butanoic acid | 107-92-6 | 814 | 790 | Pino, J.A.; Mesa, J.; Muñoz, Y.; Martí, M.P.; Marbot, R., Volatile components from mango (Mangifera indica L.) cultivars, J. Agric. Food Chem., 2005, 53, 6, 2213-2223, https://doi.org/10.1021/jf0402633 . |
| (*R,S*)-2,3-butanediol* | 513-85-9 | 794 | 784 | Xu, L.-L.; Han, T.; Wu, J.-Z.; Zhang, Q.-Y.; Zhang, H.; Huang, B.-K.; Rahman, K., Comparative research of chemical constituents, antifungal and antitumor properties of ether extracts of Panax ginseng and its endophytic fungus, 2012, retrieved from http://www.thefreelibrary.com/Comparative .... |
| 2-hexanone | 591-78-6 | 796 | 792 | Mildner-Szkudlarz, S.; Jelen, H.H., The potential of different techniques for volatile compounds analysis coupled with PCA for the detection of the adulteration of olive oil with hazelnut oil, Food Chem., 2008, 110, 3, 751-761, https://doi.org/10.1016/j.foodchem.2008.02.053 . |
| 3-hexanol | 623-37-0 | 805 | 802 | Boué, S.M.; Shih, B.Y.; Carter-Wientjes, C.H.; Cleveland, T.E., Identification of volatile compounds in soybean at various developmental stages using solid phase microextraction, J. Agric. Food Chem., 2003, 51, 17, 4873-4876, https://doi.org/10.1021/jf030051q . |
| RI800* |  | 800 |  | - |
| 2-hexanol | 626-93-7 | 815 | 811 | Boylston, T.D.; Viniyard, B.T., Isolation of volatile flavor compounds from peanut butter using purge-and-trap technique in Instrumental Methods in Food and Beverage Analysis, D. Wetzel and G. Charalambous, ed(s)., 1998, 225-243. |
| hexanal | 66-25-1 | 801 | 800 | Pino, J.A.; Mesa, J.; Muñoz, Y.; Martí, M.P.; Marbot, R., Volatile components from mango (Mangifera indica L.) cultivars, J. Agric. Food Chem., 2005, 53, 6, 2213-2223, https://doi.org/10.1021/jf0402633 . |
| ethyl butanoate* | 105-54-4 | 805 | 802 | Pino, J.A.; Mesa, J.; Muñoz, Y.; Martí, M.P.; Marbot, R., Volatile components from mango (Mangifera indica L.) cultivars, J. Agric. Food Chem., 2005, 53, 6, 2213-2223, https://doi.org/10.1021/jf0402633 . |
| butyl acetate* | 123-86-4 | 814 | 812 | Pino, J.A.; Mesa, J.; Muñoz, Y.; Martí, M.P.; Marbot, R., Volatile components from mango (Mangifera indica L.) cultivars, J. Agric. Food Chem., 2005, 53, 6, 2213-2223, https://doi.org/10.1021/jf0402633 . |
| ethyl lactate* | 97-64-3 | 817 | 813 | Ansorena, D.; Gimeno, O.; Astiasarán, I.; Bello, J., Analysis of volatile compounds by GC-MS of a dry fermented sausage: chorizo de Pamplona, Food Res. Int., 2001, 34, 1, 67-75, https://doi.org/10.1016/S0963-9969(00)00133-2 . |
| 3-methyl butyric acid | 503-74-2 | 835 | 834 | Pino, J.A.; Mesa, J.; Muñoz, Y.; Martí, M.P.; Marbot, R., Volatile components from mango (Mangifera indica L.) cultivars, J. Agric. Food Chem., 2005, 53, 6, 2213-2223, https://doi.org/10.1021/jf0402633 . |
| 3-furaldehyde | 498-60-2 | 835 | 837 | Bredie, W.L.P.; Mottram, D.S.; Guy, R.C.E., Effect of temperature and pH on the generation of flavor volatiles in extrusion cooking of wheat flour, J. Agric. Food Chem., 2002, 50, 5, 1118-1125, https://doi.org/10.1021/jf0111662 . |
| 2-(methylthio)ethanol | 5271-38-5 | 842 | 838 | Boulanger, R.; Chassagne, D.; Crouzet, J., Free and bound flavour components of amazonian fruits. 1: Bacuri, Flavour Fragr. J., 1999, 14, 5, 303-311, https://doi.org/10.1002/(SICI)1099-1026(199909/10)14:5<303::AID-FFJ834>3.0.CO;2-C . |
| 3-methyl-1-pentanol* | 589-35-5 | 846 | 843 | Jalali-Heravi, M.; Zekavat, B.; Sereshti, H., Characterization of essential oil components of Iranian geranium oil using gas chromatography-mass spectrometry combined with chemometric resolution techniques, J. Chromatogr. A, 2006, 1114, 1, 154-163, https://doi.org/10.1016/j.chroma.2006.02.034 . |
| 3-methyl butanoic acid | 503-74-2 | 853 | 845 | Ansorena, D.; Gimeno, O.; Astiasarán, I.; Bello, J., Analysis of volatile compounds by GC-MS of a dry fermented sausage: chorizo de Pamplona, Food Res. Int., 2001, 34, 1, 67-75, https://doi.org/10.1016/S0963-9969(00)00133-2 . |
| ethyl 2-methylbutanoate | 7452-79-1 | 852 | 842 | Pino, J.A.; Mesa, J.; Muñoz, Y.; Martí, M.P.; Marbot, R., Volatile components from mango (Mangifera indica L.) cultivars, J. Agric. Food Chem., 2005, 53, 6, 2213-2223, https://doi.org/10.1021/jf0402633 . |
| ethyl 3-methylbutanoate | 108-64-5 | 855 | 856 | Pino, J.A.; Mesa, J.; Muñoz, Y.; Martí, M.P.; Marbot, R., Volatile components from mango (Mangifera indica L.) cultivars, J. Agric. Food Chem., 2005, 53, 6, 2213-2223, https://doi.org/10.1021/jf0402633 . |
| 2-methyl butanoic acid | 116-53-0 | 873 | 846 | Pino, J.A.; Mesa, J.; Muñoz, Y.; Martí, M.P.; Marbot, R., Volatile components from mango (Mangifera indica L.) cultivars, J. Agric. Food Chem., 2005, 53, 6, 2213-2223, https://doi.org/10.1021/jf0402633 . |
| RI837* |  | 837 |  | - |
| ethylbenzene | 100-41-4 | 867 | 868 | Pino, J.A.; Mesa, J.; Muñoz, Y.; Martí, M.P.; Marbot, R., Volatile components from mango (Mangifera indica L.) cultivars, J. Agric. Food Chem., 2005, 53, 6, 2213-2223, https://doi.org/10.1021/jf0402633 . |
| 1-hexanol | 111-27-3 | 870 | 876 | Boué, S.M.; Shih, B.Y.; Carter-Wientjes, C.H.; Cleveland, T.E., Identification of volatile compounds in soybean at various developmental stages using solid phase microextraction, J. Agric. Food Chem., 2003, 51, 17, 4873-4876, https://doi.org/10.1021/jf030051q . |
| p-xylene | 106-42-3 | 871 | 883 | Pérez-Parajón, J.M.; Santiuste, J.M.; Takács, J.M., Sensitivity of the methylbenzenes and chlorobenzenes retention index to column temperature, stationary phase polarity, and number and chemical nature of substituents, J. Chromatogr. A, 2004, 1048, 2, 223-232, https://doi.org/10.1016/j.chroma.2004.07.028 . |
| isoamyl acetate* | 123-92-2 | 880 | 876 | Pino, J.A.; Mesa, J.; Muñoz, Y.; Martí, M.P.; Marbot, R., Volatile components from mango (Mangifera indica L.) cultivars, J. Agric. Food Chem., 2005, 53, 6, 2213-2223, https://doi.org/10.1021/jf0402633 . |
| 2-methyl-1-butyl acetate | 624-41-9 | 882 | 880 | Pino, J.A.; Mesa, J.; Muñoz, Y.; Martí, M.P.; Marbot, R., Volatile components from mango (Mangifera indica L.) cultivars, J. Agric. Food Chem., 2005, 53, 6, 2213-2223, https://doi.org/10.1021/jf0402633 . |
| 1-(methylthio)-pentane | 1741-83-9 | 883 | 900 | Sylla, K.S.B.; Berge, J.-P.; Prost, C.; Musabyemariya, B.; Seydi, M., Sensory and aromatic characteristics of tongue sole by-products hydrolysates (Cynoglossus senegalensis), Microbiologie et Hygiene Alimentarie, 2009, 21, 60, 35-43. |
| 3-heptanone | 106-35-4 | 886 | 888 | Sotomayor, J.A.; Martínez, R.M.; García, A.J.; Jordán, M.J., Thymus zygis Subsp. Gracilis: watering level effect on phytomass production and essential oil quality, J. Agric. Food Chem., 2004, 52, 17, 5418-5424, https://doi.org/10.1021/jf0496245 . |
| pentanoic acid | 109-52-4 | 909 | 887 | Mildner-Szkudlarz, S.; Jelen, H.H., The potential of different techniques for volatile compounds analysis coupled with PCA for the detection of the adulteration of olive oil with hazelnut oil, Food Chem., 2008, 110, 3, 751-761, https://doi.org/10.1016/j.foodchem.2008.02.053 . |
| acetoin acetate | 4906-24-5 | 894 | 888 | Tret'yakov, K.V., Retention Data. NIST Mass Spectrometry Data Center., NIST Mass Spectrometry Data Center, 2008. |
| 2-heptanone* | 110-43-0 | 892 | 893 | Flamini, G.; Tebano, M.; Cioni, P.L.; Bagci, Y.; Dural, H.; Ertugrul, K.; Uysal, T.; Savran, A., A multivariate statistical approach to Centaurea classification using essential oil composition data of some species from Turkey, Pl. Syst. Evol., 2006, 261, 1-4, 217-228, https://doi.org/10.1007/s00606-006-0448-3 . |
| 2-hydroxy-3-hexanone* | 54073-43-7 | 899 | 896 | [Andriamaharavo, N.R.](https://webbook.nist.gov/cgi/cbook.cgi?Author=Andriamaharavo%2C+N.R.&Units=SI&Mask=2000), Retention Data. NIST Mass Spectrometry Data Center., NIST Mass Spectrometry Data Center, 2014. |
| styrene | 100-42-5 | 897 | 890 | Pino, J.A.; Mesa, J.; Muñoz, Y.; Martí, M.P.; Marbot, R., Volatile components from mango (Mangifera indica L.) cultivars, J. Agric. Food Chem., 2005, 53, 6, 2213-2223, https://doi.org/10.1021/jf0402633 . |
| cyclohexanone | 108-94-1 | 895 | 895 | Pino, J.A.; Mesa, J.; Muñoz, Y.; Martí, M.P.; Marbot, R., Volatile components from mango (Mangifera indica L.) cultivars, J. Agric. Food Chem., 2005, 53, 6, 2213-2223, https://doi.org/10.1021/jf0402633 . |
| propyl butyrate* | 105-66-8 | 901 | 896 | Pino, J.A.; Mesa, J.; Muñoz, Y.; Martí, M.P.; Marbot, R., Volatile components from mango (Mangifera indica L.) cultivars, J. Agric. Food Chem., 2005, 53, 6, 2213-2223, https://doi.org/10.1021/jf0402633 . |
| heptanal | 111-71-7 | 904 | 899 | Pino, J.A.; Mesa, J.; Muñoz, Y.; Martí, M.P.; Marbot, R., Volatile components from mango (Mangifera indica L.) cultivars, J. Agric. Food Chem., 2005, 53, 6, 2213-2223, https://doi.org/10.1021/jf0402633 . |
| propyl butanoate | 105-66-8 | 911 | 896 | Isidorov, V.; Purzynska, A.; Modzelewska, A.; Serowiecka, M., Distribution coefficients of aliphatic alcohols, carbonyl compounds and esters between air and Carboxen/polydimethylsiloxane fiber coating, Anal. Chim. Acta., 2006, 560, 1-2, 103-109, https://doi.org/10.1016/j.aca.2005.12.043 . |
| butyrolactone | 96-48-0 | 917 | 915 | Pino, J.A.; Mesa, J.; Muñoz, Y.; Martí, M.P.; Marbot, R., Volatile components from mango (Mangifera indica L.) cultivars, J. Agric. Food Chem., 2005, 53, 6, 2213-2223, https://doi.org/10.1021/jf0402633 . |
| anisole* | 100-66-3 | 922 | 918 | Isidorov, V.; Purzynska, A.; Modzelewska, A.; Serowiecka, M., Distribution coefficients of aliphatic alcohols, carbonyl compounds and esters between air and Carboxen/polydimethylsiloxane fiber coating, Anal. Chim. Acta., 2006, 560, 1-2, 103-109, https://doi.org/10.1016/j.aca.2005.12.043 . |
| dimethyl sulfone | 67-71-0 | 918 | 914 | Andriamaharavo, N.R., Retention Data. NIST Mass Spectrometry Data Center., NIST Mass Spectrometry Data Center, 2014. |
| RI928* |  | 929 |  | - |
| citronellene | 2436-90-0 | 933 | 939 | Diaz, A.; Kite, G.C., A comparison of the pollination ecology of Arum maculatum and A. italicum in England, Watsonia, 2002, 24, 171-181. |
| RI935* |  | 935 |  | - |
| α-pinene | 80-56-8 | 939 | 940 | Jalali-Heravi, M.; Zekavat, B.; Sereshti, H., Characterization of essential oil components of Iranian geranium oil using gas chromatography-mass spectrometry combined with chemometric resolution techniques, J. Chromatogr. A, 2006, 1114, 1, 154-163, https://doi.org/10.1016/j.chroma.2006.02.034 . |
| RI948* |  | 947 |  | - |
| propyl 2-methylbutanoate | 37064-20-3 | 953 | 944 | Andriamaharavo, N.R., Retention Data. NIST Mass Spectrometry Data Center., NIST Mass Spectrometry Data Center, 2014. |
| propyl 3-methylbutanoate | 557-00-6 | 952 | 951 | Andriamaharavo, N.R., Retention Data. NIST Mass Spectrometry Data Center., NIST Mass Spectrometry Data Center, 2014. |
| 6-methyl-2-heptanone | 928-68-7 | 958 | 957 | Sylla, K.S.B.; Berge, J.-P.; Prost, C.; Musabyemariya, B.; Seydi, M., Sensory and aromatic characteristics of tongue sole by-products hydrolysates (Cynoglossus senegalensis), Microbiologie et Hygiene Alimentarie, 2009, 21, 60, 35-43. |
| propylbenzene | 103-56-1 | 959 | 954 | Isidorov, V.; Jdanova, M., Volatile organic compounds from leaves litter, Chemosphere, 2002, 48, 9, 975-979, https://doi.org/10.1016/S0045-6535(02)00074-7 . |
| benzaldehyde | 100-52-7 | 965 | 961 | Pino, J.A.; Mesa, J.; Muñoz, Y.; Martí, M.P.; Marbot, R., Volatile components from mango (Mangifera indica L.) cultivars, J. Agric. Food Chem., 2005, 53, 6, 2213-2223, https://doi.org/10.1021/jf0402633 . |
| 1-heptanol | 111-70-6 | 970 | 969 | Pino, J.A.; Mesa, J.; Muñoz, Y.; Martí, M.P.; Marbot, R., Volatile components from mango (Mangifera indica L.) cultivars, J. Agric. Food Chem., 2005, 53, 6, 2213-2223, https://doi.org/10.1021/jf0402633 . |
| dimethyl trisulfide* | 3658-80-8 | 978 | 977 | Andriamaharavo, N.R., Retention Data. NIST Mass Spectrometry Data Center., NIST Mass Spectrometry Data Center, 2014. |
| 1-octen-3-one* | 43212-99-6 | 978 | 979 | Fritsch, H.T.; Schieberle, P., Identification based on quantitative measurements and aroma recombination of the character impact odorants in a Bavarian Pilsner-type beer, J. Agric. Food Chem., 2005, 53, 19, 7544-7551, https://doi.org/10.1021/jf051167k . |
| phenol* | 108-95-2 | 981 | 984 | Nawrath, T.; Mgode, G.F.; Weetjens, B.; Kaufmann, S.H.E.; Schulz, S., The volatiles of pathogenic and nonpathogenic mycobacteria aand related bacteria, Beilstein J. Org. Chem., 2012, 8, 290-297, https://doi.org/10.3762/bjoc.8.31 . |
| 1-octen-3-ol* | 3391-86-4 | 982 | 978 | Pino, J.A.; Mesa, J.; Muñoz, Y.; Martí, M.P.; Marbot, R., Volatile components from mango (Mangifera indica L.) cultivars, J. Agric. Food Chem., 2005, 53, 6, 2213-2223, https://doi.org/10.1021/jf0402633 . |
| hexanoic acid* | 142-62-1 | 983 | 981 | Pino, J.A.; Mesa, J.; Muñoz, Y.; Martí, M.P.; Marbot, R., Volatile components from mango (Mangifera indica L.) cultivars, J. Agric. Food Chem., 2005, 53, 6, 2213-2223, https://doi.org/10.1021/jf0402633 . |
| 6-methyl-5-heptene-2-one* | 110-93-0 | 989 | 985 | Pino, J.A.; Mesa, J.; Muñoz, Y.; Martí, M.P.; Marbot, R., Volatile components from mango (Mangifera indica L.) cultivars, J. Agric. Food Chem., 2005, 53, 6, 2213-2223, https://doi.org/10.1021/jf0402633 . |
| RI998* |  | 997 |  | - |
| butyl butanoate | 109-21-7 | 998 | 994 | Pino, J.A.; Mesa, J.; Muñoz, Y.; Martí, M.P.; Marbot, R., Volatile components from mango (Mangifera indica L.) cultivars, J. Agric. Food Chem., 2005, 53, 6, 2213-2223, https://doi.org/10.1021/jf0402633 . |
| ethyl hexanoate* | 123-66-0 | 1001 | 996 | Pino, J.A.; Mesa, J.; Muñoz, Y.; Martí, M.P.; Marbot, R., Volatile components from mango (Mangifera indica L.) cultivars, J. Agric. Food Chem., 2005, 53, 6, 2213-2223, https://doi.org/10.1021/jf0402633 . |
| decane | 124-18-5 | 1000 | 1000 | - |
| octanal* | 123-13-0 | 1005 | 1001 | Pino, J.A.; Mesa, J.; Muñoz, Y.; Martí, M.P.; Marbot, R., Volatile components from mango (Mangifera indica L.) cultivars, J. Agric. Food Chem., 2005, 53, 6, 2213-2223, https://doi.org/10.1021/jf0402633 . |
| (*Z*)-3-hexen-1-yl acetate | 3681-71-8 | 1009 | 1007 | Isidorov, V.A.; Krajewska, U.; Dubis, E.N.; Jdanova, M.A., Partition coefficients of alkyl aromatic hydrocarbons and esters in a hexane-acetonitrile system, J. Chromatogr. A, 2001, 923, 1-2, 127-136, https://doi.org/10.1016/S0021-9673(01)00929-3 . |
| hexyl acetate | 142-92-7 | 1014 | 1014 | Mildner-Szkudlarz, S.; Jelen, H.H., The potential of different techniques for volatile compounds analysis coupled with PCA for the detection of the adulteration of olive oil with hazelnut oil, Food Chem., 2008, 110, 3, 751-761, https://doi.org/10.1016/j.foodchem.2008.02.053 . |
| 2-ethyl-1-hexanol* | 104-76-7 | 1030 | 1028 | Boué, S.M.; Shih, B.Y.; Carter-Wientjes, C.H.; Cleveland, T.E., Identification of volatile compounds in soybean at various developmental stages using solid phase microextraction, J. Agric. Food Chem., 2003, 51, 17, 4873-4876, https://doi.org/10.1021/jf030051q . |
| limonene | 138-86-3 | 1026 | 1035 | Jalali-Heravi, M.; Zekavat, B.; Sereshti, H., Characterization of essential oil components of Iranian geranium oil using gas chromatography-mass spectrometry combined with chemometric resolution techniques, J. Chromatogr. A, 2006, 1114, 1, 154-163, https://doi.org/10.1016/j.chroma.2006.02.034 . |
| benzyl alcohol | 100-51-6 | 1039 | 1033 | Pino, J.A.; Mesa, J.; Muñoz, Y.; Martí, M.P.; Marbot, R., Volatile components from mango (Mangifera indica L.) cultivars, J. Agric. Food Chem., 2005, 53, 6, 2213-2223, https://doi.org/10.1021/jf0402633 . |
| 1-methyl-2-pyrrolidinone | 872-50-4 | 1043 | 1045 | Leffingwell, J.C.; Alford, E.D., Volatile constituents of Perique tobacco, Electron. J. Environ. Agric. Food Chem., 2005, 4, 2, 899-915. |
| butanoic acid, 3-methylbutyl ester | 106-27-4 | 1057 | 1064 | Pino, J.A.; Mesa, J.; Muñoz, Y.; Martí, M.P.; Marbot, R., Volatile components from mango (Mangifera indica L.) cultivars, J. Agric. Food Chem., 2005, 53, 6, 2213-2223, https://doi.org/10.1021/jf0402633 . |
| γ-caprolactone | 0695-06-07 | 1058 | 1056 | Pino, J.A.; Mesa, J.; Muñoz, Y.; Martí, M.P.; Marbot, R., Volatile components from mango (Mangifera indica L.) cultivars, J. Agric. Food Chem., 2005, 53, 6, 2213-2223, https://doi.org/10.1021/jf0402633 . |
| 1-octen-2-ol | 22104-78-5 | 1069 | 1067 | Thakeow, P.; Angeli, S.; Weissbecker, B.; Schutz, S., Antennal and behavioral responses of Cis boleti to fungal odor of Trametes gibbosa, Chem. Senses, 2008, 33, 4, 379-387, https://doi.org/10.1093/chemse/bjn005 . |
| 1-octanol | 111-87-5 | 1068 | 1070 | Pino, J.A.; Mesa, J.; Muñoz, Y.; Martí, M.P.; Marbot, R., Volatile components from mango (Mangifera indica L.) cultivars, J. Agric. Food Chem., 2005, 53, 6, 2213-2223, https://doi.org/10.1021/jf0402633 . |
| p-cresol | 106-44-5 | 1076 | 1070 | Bozi, J.; Czagany, Z.; Meszaros, E.; Blazso, M., Thermal decomposition of flame retarded polycarbonates, J. Anal. Appl. Pyrolysis, 2007, 79, 1-2, 337-345, https://doi.org/10.1016/j.jaap.2007.01.001 . |
| 2-nonanone | 821-55-6 | 1094 | 1089 | Pino, J.A.; Marquez, E.; Quijano, C.E.; Castro, D., Volatile compounds in noni (Morinda citrifolia L.) at two ripening stages, Ciencia e Technologia de Alimentos, 2010, 30, 1, 183-187, https://doi.org/10.1590/S0101-20612010000100028 . |
| ethyl 2,4-hexadienoate | 2396-84-1 | 1100 | 1093 | Pino, J.A.; Marquez, E.; Quijano, C.E.; Castro, D., Volatile compounds in noni (Morinda citrifolia L.) at two ripening stages, Ciencia e Technologia de Alimentos, 2010, 30, 1, 183-187, https://doi.org/10.1590/S0101-20612010000100028 . |
| undecane | 1120-21-4 | 1102 | 1100 | - |
| linalool | 78-70-6 | 1102 | 1106 | Jalali-Heravi, M.; Zekavat, B.; Sereshti, H., Characterization of essential oil components of Iranian geranium oil using gas chromatography-mass spectrometry combined with chemometric resolution techniques, J. Chromatogr. A, 2006, 1114, 1, 154-163, https://doi.org/10.1016/j.chroma.2006.02.034 . |
| nonanal* | 124-19-6 | 1106 | 1103 | Pino, J.A.; Marquez, E.; Quijano, C.E.; Castro, D., Volatile compounds in noni (Morinda citrifolia L.) at two ripening stages, Ciencia e Technologia de Alimentos, 2010, 30, 1, 183-187, https://doi.org/10.1590/S0101-20612010000100028 . |
| phenethyl alcohol | 60-12-8 | 1118 | 1121 | Jalali-Heravi, M.; Zekavat, B.; Sereshti, H., Characterization of essential oil components of Iranian geranium oil using gas chromatography-mass spectrometry combined with chemometric resolution techniques, J. Chromatogr. A, 2006, 1114, 1, 154-163, https://doi.org/10.1016/j.chroma.2006.02.034 . |
| 1,3-dimethoxy-benzene | 151-10-0 | 1172 | 1182 | Tret'yakov, K.V., Retention Data. NIST Mass Spectrometry Data Center., NIST Mass Spectrometry Data Center, 2008. |
| diethyl butanedioate | 123-25-1 | 1180 | 1182 | Demyttenaere, J.C.R.; Dagher, C.; Sandra, P.; Kallithraka, S.; Verhé, R.; de Kimpe, N., Flavour analysis of Greek white wine by solid-phase microextraction-capillary gas chromatography-mass spectrometry, J. Chromatogr. A, 2003, 985, 1-2, 233-246, https://doi.org/10.1016/S0021-9673(02)01467-X . |
| 2-(2-butoxyethoxy)-ethanol | 112-34-5 | 1191 | 1192 | Dallüge, J.; van Stee, L.L.P.; Xu, X.; Williams, J.; Beens, J.; Vreuls, R.J.J.; Brinkman, U.A.Th., Unravelling the composition of very complex samples by comprehensive gas chromatography coupled to time-of-flight mass spectrometry. Cigarette smoke, J. Chromatogr. A, 2002, 974, 1-2, 169-184, https://doi.org/10.1016/S0021-9673(02)01384-5 . |
| 2-decanone | 693-54-9 | 1193 | 1193 | Mildner-Szkudlarz, S.; Jelen, H.H., The potential of different techniques for volatile compounds analysis coupled with PCA for the detection of the adulteration of olive oil with hazelnut oil, Food Chem., 2008, 110, 3, 751-761, https://doi.org/10.1016/j.foodchem.2008.02.053 . |
| ethyl octanoate* | 106-32-1 | 1197 | 1196 | Pino, J.A.; Mesa, J.; Muñoz, Y.; Martí, M.P.; Marbot, R., Volatile components from mango (Mangifera indica L.) cultivars, J. Agric. Food Chem., 2005, 53, 6, 2213-2223, https://doi.org/10.1021/jf0402633 . |
| α-terpinol | 98-55-5 | 1197 | 1200 | Jalali-Heravi, M.; Zekavat, B.; Sereshti, H., Characterization of essential oil components of Iranian geranium oil using gas chromatography-mass spectrometry combined with chemometric resolution techniques, J. Chromatogr. A, 2006, 1114, 1, 154-163, https://doi.org/10.1016/j.chroma.2006.02.034 . |
| dodecane | 112-40-3 | 1200 | 1200 | - |
| methyl salicylate | 119-36-8 | 1198 | 1190 | Pino, J.A.; Mesa, J.; Muñoz, Y.; Martí, M.P.; Marbot, R., Volatile components from mango (Mangifera indica L.) cultivars, J. Agric. Food Chem., 2005, 53, 6, 2213-2223, https://doi.org/10.1021/jf0402633 . |
| decanal* | 112-31-2 | 1207 | 1205 | Pino, J.A.; Mesa, J.; Muñoz, Y.; Martí, M.P.; Marbot, R., Volatile components from mango (Mangifera indica L.) cultivars, J. Agric. Food Chem., 2005, 53, 6, 2213-2223, https://doi.org/10.1021/jf0402633 . |
| octyl acetate | 112-14-1 | 1211 | 1211 | Pino, J.A.; Mesa, J.; Muñoz, Y.; Martí, M.P.; Marbot, R., Volatile components from mango (Mangifera indica L.) cultivars, J. Agric. Food Chem., 2005, 53, 6, 2213-2223, https://doi.org/10.1021/jf0402633 . |
| β-cyclocitral | 432-25-7 | 1229 | 1220 | Pino, J.A.; Mesa, J.; Muñoz, Y.; Martí, M.P.; Marbot, R., Volatile components from mango (Mangifera indica L.) cultivars, J. Agric. Food Chem., 2005, 53, 6, 2213-2223, https://doi.org/10.1021/jf0402633 . |
| dimethyl tetrasulfide | 5756-24-1 | 1228 | 1215 | Bonaiti, C.; Irlinger, F.; Spinnler, H.E.; Engel, E., An iterative sensory procedure to select odor-active associations in complex consortia of microorganisms: application to the construction of a cheese model, J. Dairy Sci., 2005, 88, 5, 1671-1684, https://doi.org/10.3168/jds.S0022-0302(05)72839-3 . |
| benzothiazole | 95-16-9 | 1233 | 1236 | Boylston, T.D.; Viniyard, B.T., Isolation of volatile flavor compounds from peanut butter using purge-and-trap technique in Instrumental Methods in Food and Beverage Analysis, D. Wetzel and G. Charalambous, ed(s)., 1998, 225-243. |
| quinoline | 91-22-5 | 1248 | 1242 | Du, Z.; Clery, R.; Hammond, C.J., Volatile organic nitrogen-containing constituents in ambrette seed Abelmoschus moschatus Medik (Malvaceae), J. Agric. Food Chem., 2008, 56, 16, 7388-7392, https://doi.org/10.1021/jf800958d . |
| 2-decenal | 3913-81-3 | 1261 | 1261 | Pino, J.A.; Mesa, J.; Muñoz, Y.; Martí, M.P.; Marbot, R., Volatile components from mango (Mangifera indica L.) cultivars, J. Agric. Food Chem., 2005, 53, 6, 2213-2223, https://doi.org/10.1021/jf0402633 . |
| 3-undecanone | 2216-87-7 | 1290 | 1283 | Lazarevic, J.; Radulovic, N.; Palic, R.; Zlatkovic, B., Chemical Analusis of volatile constituents of Berula erecta (Hudson) Coville subsp. erecta (Apiaceae) from Serbia, J. Essential Oil. Res., 2010, 22, 3, 153-156, https://doi.org/10.1080/10412905.2010.9700290 . |
| anethole | 4180-23-8 | 1292 | 1283 | Pino, J.A.; Mesa, J.; Muñoz, Y.; Martí, M.P.; Marbot, R., Volatile components from mango (Mangifera indica L.) cultivars, J. Agric. Food Chem., 2005, 53, 6, 2213-2223, https://doi.org/10.1021/jf0402633 . |
| vitispirane | 65416-59-3 | 1291 | 1283 | Demyttenaere, J.C.R.; Dagher, C.; Sandra, P.; Kallithraka, S.; Verhé, R.; de Kimpe, N., Flavour analysis of Greek white wine by solid-phase microextraction-capillary gas chromatography-mass spectrometry, J. Chromatogr. A, 2003, 985, 1-2, 233-246, https://doi.org/10.1016/S0021-9673(02)01467-X . |
| bornyl acetate | 76-49-3 | 1288 | 1288 | Lopes, D.; Strobl, H.; Kolodziejczyk, P., 14-Methylpentadecano-15-lactone (Muscolide): a new macrocyclic lactone from the oil of Angelica archangelica L., Chemistry and Biodiversity, 2004, 1, 12, 1880-1887, https://doi.org/10.1002/cbdv.200490144 . |
| 2-undecanone | 0112-12-9 | 1294 | 1293 | Kallio, M.; Jussila, M.; Rissanen, T.; Anttila, P.; Hartonen, K.; Reissell, A.; Vreuls, R.; Adahchour, M.; Hyotylainen, T., Comprehensive two-dimensional gas chromatography coupled to time-of-flight mass spectrometry in the identification of organic compounds in atmospheric aerosols from coniferous forest, J. Chromatogr. A, 2006, 1125, 2, 234-243, https://doi.org/10.1016/j.chroma.2006.05.050 . |
| tridecane | 629-50-5 | 1299 | 1300 | - |
| indole | 120-72-9 | 1301 | 1299 | Miyazawa, M.; Marumoto, S.; Kobayashi, T.; Yoshida, S.; Utsumi, Y., Determination of characteristic components in essential oils from Wisteria braphybotrys using gas chromatography - olfactometry incremental dilution technique, Rec. Nat. Prod., 2011, 5, 3, 221-227. |
| undecanal | 112-44-7 | 1308 | 1306 | Pino, J.A.; Mesa, J.; Muñoz, Y.; Martí, M.P.; Marbot, R., Volatile components from mango (Mangifera indica L.) cultivars, J. Agric. Food Chem., 2005, 53, 6, 2213-2223, https://doi.org/10.1021/jf0402633 . |
| quinaldine | 91-63-4 | 1318 | 1312 | Rostad, C.E.; Pereira, W.E., Kovats and Lee retention indices determined by gas chromatography/mass spectrometry for organic compounds of environmental interest, J. Hi. Res. Chromatogr. Chromatogr. Comm., 1986, 9, 6, 328-334, https://doi.org/10.1002/jhrc.1240090603 . |
| γ-nonalactone | 104-61-0 | 1368 | 1363 | Lozano P.R.; Drake M.; Benitez D.; Cadwallader K.R., Instrumental and sensory characterization of heat-induced odorants in aseptically packaged soy milk, J. Agric. Food Chem., 2007, 55, 8, 3018-3026, https://doi.org/10.1021/jf0631225 . |
| α-copaene | 3856-25-5 | 1387 | 1394 | Jalali-Heravi, M.; Zekavat, B.; Sereshti, H., Characterization of essential oil components of Iranian geranium oil using gas chromatography-mass spectrometry combined with chemometric resolution techniques, J. Chromatogr. A, 2006, 1114, 1, 154-163, https://doi.org/10.1016/j.chroma.2006.02.034 . |
| ethyl decanoate | 110-38-3 | 1395 | 1397 | Pino, J.A.; Mesa, J.; Muñoz, Y.; Martí, M.P.; Marbot, R., Volatile components from mango (Mangifera indica L.) cultivars, J. Agric. Food Chem., 2005, 53, 6, 2213-2223, https://doi.org/10.1021/jf0402633 . |
| tetradecane | 629-59-4 | 1399 | 1400 | - |
| dodecanal | 112-54-9 | 1411 | 1409 | Ogunwande, I.A.; Flamini, G.; Cioni, P.L.; Omikorede, O.; Azeez, R.A.; Ayodele, A.A.; Kamil, Y.O., Aromatic plants growing in Nigeria: essential oil constituents of Cassia alata (Linn.) Roxb. and Helianthus annuus L., Rec. Nat. Prod., 2010, 4, 4, 211-217. |
| caryophyllene | 87-44-5 | 1435 | 1444 | Jalali-Heravi, M.; Zekavat, B.; Sereshti, H., Characterization of essential oil components of Iranian geranium oil using gas chromatography-mass spectrometry combined with chemometric resolution techniques, J. Chromatogr. A, 2006, 1114, 1, 154-163, https://doi.org/10.1016/j.chroma.2006.02.034 . |
| α-bergamotene | 13474-59-4 | 1445 | 1440 | Bagci, E.; Yazgin, A.; Hayta, S.; Cakilcioglu, U., Composition of the essential oil of Teucrium chamaedrys L. (Lamiaceae) from Turkey, J. Med. Plants Res., 2010, 4, 23, 2588-2590. |
| geranyl acetone | 68228-05-07 | 1456 | 1458 | Alissandrakis E.; Tarantilis P.A.; Harizanis P.C.; Polissiou M., Comparison of the volatile composition in thyme honeys from several origins in Greece, J. Agric. Food Chem., 2007, 55, 20, 8152-8157, https://doi.org/10.1021/jf071442y . |
| trans-β-ionone | 79-77-6 | 1495 | 1992 | Javidnia, K.; Miri, R.; Banani, A., Volatile oil constituents of Haplophyllum tuberculatum (Forssk.) A. Juss. (Rutaceae) from Iran, J. Essent. Oil Res., 2006, 18, 4, 355-356, https://doi.org/10.1080/10412905.2006.9699111 . |
| pentadecane | 629-62-9 | 1499 | 1500 | - |
| dodecanoic acid | 0143-07-07 | 1571 | 1568 | Saroglou, V.; Dorizas, N.; Kypriotakis, Z.; Skaltsa, H.D., Analysis of the essential oil composition of eight Anthemis species from Greece, J. Chromatogr. A, 2006, 1104, 1-2, 313-322, https://doi.org/10.1016/j.chroma.2005.11.087 . |
| hexadecane | 544-76-3 | 1599 | 1600 | - |
| tetradecanal | 124-25-4 | 1614 | 1611 | Saroglou, V.; Dorizas, N.; Kypriotakis, Z.; Skaltsa, H.D., Analysis of the essential oil composition of eight Anthemis species from Greece, J. Chromatogr. A, 2006, 1104, 1-2, 313-322, https://doi.org/10.1016/j.chroma.2005.11.087 . |
| isopropyl laurate | 10233-13-3 | 1627 | 1629 | Radulovic, N.; Blagojevic, P.; Palic, R., Comparative study of the leaf volatiles of Arctostaphylos uva-ursi (L.) Spreng. and Vaccinium vitis-idaea L. (Ericaceae), Molecules, 2010, 15, 9, 6168-6185, https://doi.org/10.3390/molecules15096168 . |
| benzophenone | 119-61-9 | 1644 | 1635 | Leffingwell, J.C.; Alford, E.D., Volatile constituents of Perique tobacco, Electron. J. Environ. Agric. Food Chem., 2005, 4, 2, 899-915. |
| heptadecane | 629-78-7 | 1699 | 1700 | - |
| tetradecanoic acid | 544-63-8 | 1765 | 1776 | Javidnia, K.; Miri, R.; Mehregan, I.; Sadeghpour, H., Volatile constituents of the essential oil of Nepeta ucrainica L. ssp. kopetdaghensis from Iran, Flavour Fragr. J., 2005, 20, 2, 219-221, https://doi.org/10.1002/ffj.1381 . |
| octadecane | 593-45-3 | 1798 | 1800 | - |
| hexadecanoic acid | 57-10-3 | 1963 | 1969 | Saroglou, V.; Dorizas, N.; Kypriotakis, Z.; Skaltsa, H.D., Analysis of the essential oil composition of eight Anthemis species from Greece, J. Chromatogr. A, 2006, 1104, 1-2, 313-322, https://doi.org/10.1016/j.chroma.2005.11.087 . |
