# Supplementary material for: Olfactory responses of the variegated fruit fly, Phortica variegata, an emerging vector of the zoonotic eyeworm Thelazia callipaeda, to ecologically relevant volatiles
Source: Parasit Vectors. 2025 Jun 2;18:204. doi: 10.1186/s13071-025-06850-8 (PMC12131565; doi:10.1186/s13071-025-06850-8)
Supplement: Supplementary file 5 — Additional file 5: Table S3. Results of multilevel pattern analysis for comparing antennal responses to synthetic compounds between D. melanogaster and P. variegata females and males. The P-value was adjusted (P-value adj.) using Benjamini-Hochberg adjustment. The column pattern shows which species and sex combinations showed significantly higher relative responses. [file 13071_2025_6850_MOESM5_ESM.docx]

**Table S3.** Results of multilevel pattern analysis for comparing antennal responses to synthetic compounds between *D*. *melanogaster* and *P*. *variegata* females and males. The *P*-value was adjusted (*P*-value adj.) using Benjamini-Hochberg adjustment. The column pattern shows which species and sex combinations showed significantly higher relative responses.

| compound | *D. melanogaster* | | *P. variegata* | | stat | *P*-value | *P*-value adj. | pattern |
| --- | --- | --- | --- | --- | --- | --- | --- | --- |
|  | ♀ | ♂ | ♀ | ♂ |  |  |  |  |
| (*E*)-2-hexenal | a | a | b | b | 0.878226 | <0.05 | <0.05 | *D melanogaster* |
| ethyl benzoate | a | a | b | b | 0.831814 | <0.05 | <0.05 | *D melanogaster* |
| 3-octanol | a | a | b | b | 0.809822 | <0.05 | <0.05 | *D melanogaster* |
| geosmin | a | a | b | b | 0.836567 | <0.05 | <0.05 | *D melanogaster* |
| phenylethyl alcohol | a | a | b | b | 0.886336 | <0.05 | <0.05 | *D melanogaster* |
| ethyl propionate | b | b | a | a | 0.906256 | <0.05 | <0.05 | *P. variegata* |
| propyl acetate | b | b | a | a | 0.857912 | <0.05 | <0.05 | *P. variegata* |
| butyl propionate | b | b | a | a | 0.899943 | <0.05 | <0.05 | *P. variegata* |
| anisole | b | b | a | a | 0.929341 | <0.05 | <0.05 | *P. variegata* |
| β-ionone | a | b | b | b | 0.961542 | <0.05 | <0.05 | *D. melanogaster ♀* |
| β-caryophyllene | a | b | b | b | 0.942205 | <0.05 | <0.05 | *D. melanogaster ♀* |
| α-humulene | a | b | b | b | 0.80575 | <0.05 | <0.05 | *D. melanogaster ♀* |
| caryophyllene oxide | a | b | b | b | 0.973781 | <0.05 | <0.05 | *D. melanogaster ♀* |
| geranyl acetate | a | b | b | b | 0.984999 | <0.05 | <0.05 | *D. melanogaster ♀* |
| citral | a | b | b | b | 0.993949 | <0.05 | <0.05 | *D. melanogaster ♀* |
| 2-heptanone | b | a | b | b | 0.716191 | <0.05 | <0.05 | *D. melanogaster ♂* |
| ethyl hexanoate | b | b | a | b | 0.735274 | <0.05 | <0.05 | *P.variegata ♀* |
| 1-pentanol | b | b | a | b | 0.803131 | <0.05 | <0.05 | *P.variegata ♀* |
| 3-octanone | b | b | b | a | 0.854723 | <0.05 | <0.05 | *P.variegata ♂* |
| phenol | a | b | b | a | 0.973387 | <0.05 | <0.05 | *D. melanogaster ♀+ P.variegata ♂* |
| isoamyl acetate | a | b | b | a | 0.834289 | <0.05 | <0.05 | *D. melanogaster ♂+ P.variegata ♂* |
| sulcatone | a | a | b | a | 0.851145 | <0.05 | <0.05 | *D. melanogaster+ P.variegata ♂* |
| decanal | a | b | a | a | 0.831976 | <0.05 | <0.05 | *D. melanogaster ♀ + P. variegata* |
| nonanal | a | b | a | a | 0.890268 | <0.05 | <0.05 | *D. melanogaster ♀ + P. variegata* |
