# Supplementary material for: Olfactory responses of the variegated fruit fly, Phortica variegata, an emerging vector of the zoonotic eyeworm Thelazia callipaeda, to ecologically relevant volatiles
Source: Parasit Vectors. 2025 Jun 2;18:204. doi: 10.1186/s13071-025-06850-8 (PMC12131565; doi:10.1186/s13071-025-06850-8)
Supplement: Supplementary file 6 — Additional file 6: Table S4. The composition of volatile samples tested using gas chromatography with electroantennographic recording. The relative abundance of each component was calculated as a percentage compared to the component with the highest peak area. [file 13071_2025_6850_MOESM6_ESM.docx]

**Table**  **S4**. The composition of volatile samples tested using gas chromatography with electroantennographic recording. The relative abundance of each component was calculated as a percentage compared to the component with the highest peak area.

| compound | Identification | CAS | human body (%) | red deer (%) | ACV (%) | brown bear (%) | red fox (%) |
| --- | --- | --- | --- | --- | --- | --- | --- |
| dimethyl disulfide | RI | 624-92-0 | - | - | - | - | 13.2 |
| 3-methyl-2-pentanone | RI | 565-61-7 | - | - | - | - | 0.6 |
| 2-methyl-propanoic acid | RI | 79-31-2 | 0.9 | 1.2 | 1.7 | 13.6 | 6.8 |
| 1-pentanol | ref, RI | 571-41-0 | 0.9 | 2.9 | - | 1.2 | 2.0 |
| toluene | RI | 108-88-3 | 1.2 | - | 0.6 | - | 2.3 |
| ethyl 2-methylpropanoate | RI | 97-62-1 | - | - | 0.6 | - | - |
| 2-methyl-1-propanol | RI | 110-19-0 | - | - | 10.6 | - | - |
| methyl 2-methylbutanoate | RI | 868-57-5 | - | - | - | - | 0.6 |
| (*R*,*R*)-2,3-butanediol | ref, RI | 513-85-9 | - | 4.9 | 9.4 | - | - |
| 2,3-hexanedione | RI | 3848-24-6 | - | - | - | 0.2 | - |
| butanoic acid | RI | 107-92-6 | - | 100.0 | 1.0 | - | 1.7 |
| (*R*,*S*)-2,3-butanediol | ref, RI | 513-85-9 | - | 3.9 | 5.3 | - | - |
| 2-hexanone | RI | 591-78-6 | - | - | - | - | 7.6 |
| 3-hexanol | RI | 623-37-0 | 1.7 | - | 0.3 | - | - |
| RI800 | RI | - | - | - | - | 1.4 | 5.2 |
| 2-hexanol | RI | 626-93-7 | 1.4 | - | - | - | - |
| hexanal | RI | 66-25-1 | 4.3 | - | - | - | 3.1 |
| ethyl butanoate | ref, RI | 105-54-4 | - | 1.2 | 2.8 | 1.7 | - |
| butyl acetate | RI | 123-86-4 | 1.2 | - | - | - | 0.8 |
| ethyl lactate | RI | 97-64-3 | - | - | 12.9 | - | - |
| 3-methyl butyric acid | RI | 503-74-2 | 0.2 | - | - | 27.5 | - |
| 3-furaldehyde | RI | 498-60-2 | 1.0 | - | 1.9 | 0.6 | 2.4 |
| 2-(methylthio)ethanol | RI | 5271-38-5 | - | - | - | 0.5 | - |
| 3-methyl-1-pentanol | RI | 589-35-5 | - | - | - | 0.4 | - |
| 3-methyl butanoic acid | RI | 503-74-2 | - | - | 5.3 | 4.0 | 11.2 |
| ethyl 2-methylbutanoate | RI | 7452-79-1 | - | - | 0.6 | - | - |
| ethyl 3-methylbutanoate | RI | 108-64-5 | - | - | 1.6 | - | - |
| 2-methyl butanoic acid | RI | 116-53-0 | - | 10.2 | 0.7 | 7.6 | 6.3 |
| RI837 | RI | - | - | 0.4 | - | - | - |
| ethylbenzene | RI | 100-41-4 | 0.9 | - | 0.4 | 0.9 | - |
| 1-hexanol | ref, RI | 111-27-3 | - | 2.9 | 3.6 | 10.4 | 0.4 |
| p-xylene | RI | 106-42-3 | 2.7 | - | 1.7 | 2.3 | 5.5 |
| isoamyl acetate | ref, RI | 123-92-2 | - | - | 100.0 | - | - |
| 2-methyl-1-butyl acetate | RI | 624-41-9 | - | - | 28.3 | - | - |
| 1-(methylthio)-pentane | RI | 1741-83-9 | - | - | - | - | 4.5 |
| 3-heptanone | RI | 106-35-4 | 2.1 | - | 0.4 | - | 1.0 |
| pentanoic acid | RI | 109-52-4 | - | 17.2 | - | - | 7.5 |
| RI894 | RI | - | - | - | 1.2 | - | - |
| 2-heptanone | ref, RI | 110-43-0 | 0.2 | 0.7 | - | 3.3 | - |
| 2-hydroxy-3-hexanone | RI | 54073-43-7 | - | - | - | 7.0 | - |
| styrene | RI | 100-42-5 | - | - | - | - | 1.9 |
| cyclohexanone | RI | 108-94-1 | 1.7 | - | - | - | - |
| propyl butyrate | RI | 105-66-8 | - | 0.9 | - | - | - |
| heptanal | RI | 111-71-7 | 1.2 | - | 0.7 | 0.6 | 1.0 |
| propyl butanoate | RI | 105-66-8 | - | 0.8 | - | - | - |
| butyrolactone | RI | 96-48-0 | - | - | 0.4 | - | - |
| anisole | ref, RI | 100-66-3 | - | - | - | - | 0.7 |
| dimethyl sulfone | RI | 67-71-0 | 0.6 | 0.5 | - | - | - |
| RI928 | RI | - | - | - | 0.2 | - | - |
| citronellene | RI | 2436-90-0 | - | 0.2 | - | - | - |
| RI935 | RI | - | - | - | 0.1 | - | - |
| α-pinene | RI | 80-56-8 | 1.7 | - | 0.6 | 0.8 | 3.5 |
| RI948 | RI | - | - | 0.4 | - | 0.3 | 0.8 |
| propyl 2-methylbutanoate | RI | 37064-20-3 | - | 2.0 | - | 0.3 | - |
| propyl 3-methylbutanoate | RI | 557-00-6 | - | - | - | 0.4 | - |
| 6-methyl-2-heptanone | RI | 928-68-7 | - | - | - | 0.3 | 1.0 |
| propylbenzene | RI | 103-56-1 | - | - | - | 0.2 | - |
| benzaldehyde | ref, RI | 100-52-7 | 8.2 | 1.3 | 5.9 | 2.4 | 7.0 |
| 1-heptanol | ref, RI | 111-70-6 | 1.3 | 1.5 | 0.4 | 2.1 | 2.4 |
| dimethyl trisulfide | RI | 3658-80-8 | - | - | - | 0.8 | 91.0 |
| 1-octen-3-one | RI | 43212-99-6 | - | 0.5 | - | - | - |
| phenol | ref, RI | 108-95-2 | - | - | - | 13.3 | 53.5 |
| 1-octen-3-ol | ref, RI | 3391-86-4 | - | 3.7 | - | - | - |
| hexanoic acid | RI | 142-62-1 | 1.9 | 1.0 | 0.4 | - | - |
| 6-methyl-5-heptan-2-one | ref, RI | 110-93-0 | 8.3 | 4.5 | 18.3 | 5.8 | 11.0 |
| RI998 | RI | - | - | - | - | 2.9 | - |
| butyl butanoate | RI | 109-21-7 | - | 3.0 | - | - | - |
| ethyl hexanoate | ref, RI | 123-66-0 | - | - | 10.6 | - | - |
| decane | RI | 124-18-5 | 0.2 | - | - | - | - |
| octanal | RI | 123-13-0 | 4.1 | - | 3.4 | 1.0 | 1.6 |
| (*Z*)-3-hexen-1-yl acetate | RI | 3681-71-8 | 0.4 | - | - | 0.6 | 1.2 |
| hexyl acetate | RI | 142-92-7 | - | - | 0.6 | - | - |
| 2-ethyl-1-hexanol | RI | 104-76-7 | 100.0 | 1.2 | 21.8 | 21.4 | 37.1 |
| limonene | RI | 138-86-3 | 1.6 | - | - | - | - |
| benzyl alcohol | RI | 100-51-6 | 0.8 | 2.5 | - | - | - |
| 1-methyl-2-pyrrolidinone | RI | 872-50-4 | - | - | 14.6 | - | - |
| isoamyl butanoate | RI | 106-27-4 | - | - | - | 0.2 | - |
| γ-caprolactone | RI | 0695-06-07 | - | 1.9 | - | - | - |
| 1-octen-2-ol | RI | 22104-78-5 | - | 0.7 | - | - | - |
| 1-octanol | RI | 111-87-5 | 3.6 | - | - | - | - |
| p-cresol | ref, RI | 106-44-5 | - | - | - | 80.7 | 100.0 |
| 2-nonanone | RI | 821-55-6 | - | 2.7 | - | 3.5 | - |
| ethyl 2,4-hexadienoate | RI | 2396-84-1 | - | - | 1.0 | - | - |
| undecane | RI | 1120-21-4 | 2.1 | - | 0.2 | 1.2 | - |
| linalool | ref, RI | 78-70-6 | - | 1.0 | - | 0.6 | - |
| nonanal | ref, RI | 124-19-6 | 8.7 | 0.8 | 7.3 | 2.8 | 4.8 |
| phenethyl alcohol | ref, RI | 60-12-8 | 3.0 | 5.6 | 11.5 | 2.8 | 19.5 |
| 1,3-dimethoxy-benzene | RI | 151-10-0 | - | - | - | - | 5.6 |
| diethyl butanedioate | RI | 123-25-1 | - | - | 2.1 | - | - |
| 2-(2-butoxyethoxy)-ethanol | RI | 112-34-5 | - | - | - | 0.9 | 2.7 |
| 2-decanone | RI | 693-54-9 | - | 0.6 | - | - | - |
| ethyl octanoate* | RI | 106-32-1 | - | - | 13.8 | - | - |
| α-terpinol | RI | 98-55-5 | - | - | - | 0.1 | - |
| dodecane | RI | 112-40-3 | - | - | - | - | 0.3 |
| methyl salicylate | ref, RI | 119-36-8 | 1.6 | - | - | - | - |
| decanal* | ref, RI | 112-31-2 | 20.2 | 0.9 | 26.0 | 4.6 | 6.5 |
| octyl acetate | RI | 112-14-1 | - | - | - | 0.6 | - |
| β-cyclocitral | RI | 432-25-7 | - | 0.2 | - | - | - |
| dimethyl tetrasulfide | RI | 5756-24-1 | - | - | - | 1.7 | 33.4 |
| benzothiazole | RI | 95-16-9 | 0.8 | - | 1.3 | 0.9 | 1.2 |
| quinoline | RI | 91-22-5 | - | - | - | - | 4.3 |
| (*E*)-2-decenal | RI | 3913-81-3 | - | 0.3 | - | - | - |
| 3-undecanone | RI | 2216-87-7 | - | - | - | 0.6 | - |
| anethole | RI | 4180-23-8 | - | - | - | - | 0.5 |
| vitispirane | RI | 65416-59-3 | - | - | 1.5 | - | - |
| bornyl acetate | RI | 76-49-3 | 0.8 | - | - | - | - |
| 2-undecanone | RI | 0112-12-9 | - | 0.4 | - | 3.4 | - |
| tridecane | RI | 629-50-5 | - | 0.1 | - | - | - |
| indole | ref, RI | 120-72-9 | - | - | - | 30.1 | 73.9 |
| undecanal | RI | 112-44-7 | 2.5 | - | 1.5 | 0.5 | 0.6 |
| quinaldine | RI | 91-63-4 | - | - | - | - | 1.8 |
| γ-nonalactone | RI | 104-61-0 | - | 0.4 | - | - | - |
| α-copaene | RI | 3856-25-5 | - | 0.2 | - | - | - |
| ethyl decanoate | RI | 110-38-3 | - | - | 2.1 | - | - |
| tetradecane | RI | 629-59-4 | 0.9 | 0.2 | 0.7 | 0.6 | - |
| dodecanal | RI | 112-54-9 | 1.4 | - | 1.4 | - | - |
| caryophyllene | ref, RI | 87-44-5 | - | 0.2 | - | - | - |
| α-bergamotene | RI | 13474-59-4 | - | - | - | 0.7 | - |
| geranyl acetone | ref, RI | 68228-05-07 | 30.2 | 0.5 | 17.1 | 1.6 | 4.1 |
| *trans*-β-ionone | ref, RI | 79-77-6 | - | 0.5 | - | - | - |
| pentadecane | RI | 629-62-9 | 1.3 | 0.6 | 1.1 | 0.6 | 1.3 |
| dodecanoic acid | RI | 0143-07-07 | 2.8 | - | - | - | - |
| hexadecane | RI | 544-76-3 | 1.1 | 0.5 | 2.4 | - | - |
| tetradecanal | RI | 124-25-4 | - | 0.2 | - | - | - |
| isopropyl laurate | RI | 10233-13-3 | - | - | 1.0 | - | - |
| benzophenone | RI | 119-61-9 | - | - | - | 0.5 | - |
| heptadecane | RI | 629-78-7 | - | 0.4 | 1.6 | 0.5 | 0.8 |
| tetradecanoic acid | RI | 544-63-8 | 3.2 | - | - | - | - |
| octadecane | RI | 593-45-3 | - | 0.3 | 0.8 | - | - |
| hexadecanoic acid | RI | 57-10-3 | 8.3 | - | - | - | - |
